# Effect of the Ancillary Ligand on the Performance of Heteroleptic Cu(I) Diimine Complexes as Dyes in Dye-Sensitized Solar Cells

**DOI:** 10.1021/acsaem.1c02778

**Published:** 2022-01-13

**Authors:** Daniele Franchi, Valentina Leandri, Angela Raffaella Pia Pizzichetti, Bo Xu, Yan Hao, Wei Zhang, Tamara Sloboda, Sebastian Svanström, Ute B. Cappel, Lars Kloo, Licheng Sun, James M. Gardner

**Affiliations:** † Institute of Chemistry of Organometallic Compounds (CNR-ICCOM), Via Madonna del Piano 10, 50019 Sesto Fiorentino, Italy; ‡ Division of Organic Chemistry, Centre of Molecular Devices, Department of Chemistry, KTH Royal Institute of Technology, SE-10044 Stockholm, Sweden; § Division of Applied Physical Chemistry, Centre of Molecular Devices, Department of Chemistry, KTH Royal Institute of Technology, SE-10044 Stockholm, Sweden; ∥ Division of Physical Chemistry, Centre of Molecular Devices, Department of Chemistry, Ångström Laboratory, Uppsala University, Box 523, SE-75120 Uppsala, Sweden; ⊥ Division of X-ray Photon Science, Department of Physics and Astronomy, Uppsala University, Box 516, SE-751 20 Uppsala, Sweden; # Center of Artificial Photosynthesis for Solar Fuels, School of Science, Westlake University, Hangzhou 310024, China

**Keywords:** DSSC, diimine copper(I) complexes, copper photosensitizers, in situ assembling, heteroleptic complexes, hard X-ray photoelectron spectroscopy, push−pull, density functional theory calculation

## Abstract

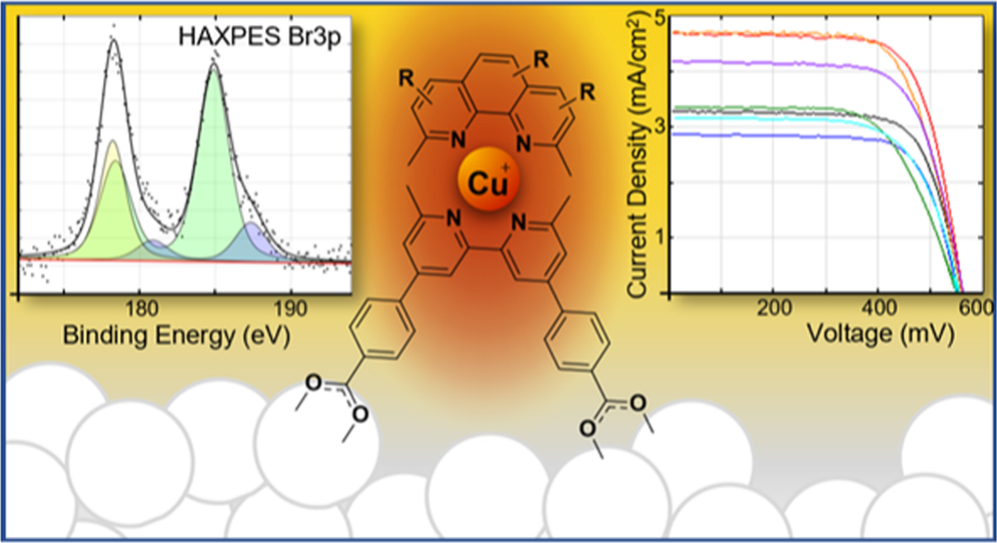

A series of heteroleptic Cu(I) diimine complexes with different ancillary ligands and 6,6′-dimethyl-2,2′-bipyridine-4,4′-dibenzoic acid (dbda) as the anchoring ligand were self-assembled on TiO_2_ surfaces and used as dyes for dye-sensitized solar cells (DSSCs). The binding to the TiO_2_ surface was studied by hard X-ray photoelectron spectroscopy for a bromine-containing complex, confirming the complex formation. The performance of all complexes was assessed and rationalized on the basis of their respective ancillary ligand. The DSSC photocurrent–voltage characteristics, incident photon-to-current conversion efficiency (IPCE) spectra, and calculated lowest unoccupied molecular orbital (LUMO) distributions collectively show a push–pull structural dye design, in which the ancillary ligand exhibits an electron-donating effect that can lead to improved solar cell performance. By analyzing the optical properties of the dyes and their solar cell performance, we can conclude that the presence of ancillary ligands with bulky substituents protects the Cu(I) metal center from solvent coordination constituting a critical factor in the design of efficient Cu(I)-based dyes. Moreover, we have identified some components in the I^–^/I_3_^–^-based electrolyte that causes dissociation of the ancillary ligand, i.e., TiO_2_ photoelectrode bleaching. Finally, the detailed studies on one of the dyes revealed an electrolyte–dye interaction, leading to a dramatic change of the dye properties when adsorbed on the TiO_2_ surface.

## Introduction

Inspired by the natural process of photosynthesis, dye-sensitized solar cells (DSSCs) have received great interest in the past 30 years.^1,2^ One of the features that distinguish DSSCs from other photovoltaic technologies is the possibility to easily tune their color, paving the way for nonconventional applications involving building integration and indoor applications.^3,4^ The reason for the simplicity of color tuning of DSSCs, as compared to other types of solar cells, resides in one of their key components: the molecular dye or photosensitizer.

In DSSCs, the dye is typically an organic, molecular compound, or a coordination complex, which is responsible for light harvesting and electron transfer into the conduction band (CB) of a semiconductor electrode (typically TiO_2_) to which it is chemically bonded. Organic dyes offer high molar extinction coefficients, important for light harvesting, and an extended structural variety that can be tuned to optimize the spectral absorption range.^5−11^ Despite the appealing properties of the organic sensitizers, it is mostly coordination complexes that have offered record performances in DSSCs.^12^ The first, and perhaps the most famous, class of metal complexes employed as dyes consists of ruthenium(II) polypyridyl complexes.^13^ Some of these compounds have shown record photon-to-current conversion efficiencies (PCEs) above 10% for more than a decade.^14,15^ Among them, the most well-known dyes are the [*cis*-(dithiocyanato)-Ru-bis(2,2′-bipyridine-4,4′-dicarboxylate)] complex, N3, and its doubly protonated tetrabutylammonium salt, N719.^16−18^ Another class of metal complexes that have been widely studied as photosensitizers is metal-based porphyrin derivatives.^19,20^ The Zn porphyrins YD2-o-C8, SM371, and SM315 are of particular interest, as they have shown superior performances to those of the Ru(II) complexes in DSSCs, exhibiting solar cell PCEs of 11.9, 12.0, and 13.0%, respectively.^21,22^ More recently, Cu(I) diimine complexes have attracted special attention.^23−25^ These complexes have been used for a variety of applications in DSSCs as hole-transporting materials (HTM), redox mediators, as well as dyes.^26−29^ The potential for combining copper(I) dyes with a wide band gap semiconductor such as TiO_2_ for light harvesting and photoconversion was first demonstrated by Sauvage and co-workers in 1994.^30,31^ Despite a growing interest in the photovoltaic application of bis(diimine)copper(I) complexes, little progress was made in the following years.^32^ In 2008, Schaffner and co-workers prepared DSSCs based on homoleptic copper(I) complexes with 6,6′-disubstituted 2,2′-bipyridines as dyes, showing promising results.^33^ The symmetrical nature of the homoleptic copper(I) complexes limits the efficiency of a preferred excited-state electron transfer into the TiO_2_ CB. Therefore, to maximize the electron injection process, heteroleptic complexes mimicking the push–pull design exploited in the design of organic dyes have been synthesized.^34−39^ However, copper(I) complexes in solution are notoriously labile and the exchange of ligands rapidly occurs. Therefore, the synthesis and study of heteroleptic copper(I) compounds can be challenging.^40−42^ One solution to this problem is to rely on bulky substituents, which prevent the formation of the corresponding homoleptic complexes, thus leading to stable heteroleptic copper(I) complexes.^43,44^ This concept has been explored by Odobel and co-workers, who prepared heteroleptic Cu(I) dyes for DSSCs showing a PCE of up to 4.7%.^45,46^ An alternative to this approach was reported by Housecroft and Constable,^47,48^ who adopted a stepwise, on-surface, self-assembly of the heteroleptic complexes by soaking a TiO_2_ substrate first in a solution of an anchoring ligand L′, and subsequently in a solution containing the homoleptic complex of copper(I) with the ancillary ligand L″, [Cu(L″)_2_]^+^. This allowed them to generate, through ligand scrambling, the desired heteroleptic complex [Cu(L′)(L″)]^+^ anchored on the semiconductor surface.^34,36^

In our previous work, we have explored the optical and electrochemical properties of a series of heteroleptic Cu(I) diimine complexes with 6,6′-dimethyl-2,2′-bipyridine-4,4′-dibenzoic acid (dbda) as an anchoring ligand and different ancillary ligands: 2,9-dimethyl-1,10-phenanthroline (dmp); 5-bromo-2,9-dimethyl-1,10-phenanthroline (Br-dmp); 2,9-dimethyl-4,7-diphenyl-1,10-phenanthroline (bcp); 2,9-di(*sec*-butyl)-3,4,7,8-tetramethyl-1,10-phenanthroline (dsbtmp); 2,2′-biquinoline (biq); and 2,9-dianisyl-1,10-phenanthroline (dap).^39^ The complexes were self-assembled on a TiO_2_ surface using the aforementioned method. Here, we continue with an investigation of the complexes as dyes for DSSCs, and we focus on rationalizing the role of the ancillary ligand with respect to the photovoltaic performance of DSSCs.

## Experimental Section

### General Information

All chemicals were purchased from Sigma-Aldrich and used as received unless noted otherwise. The ligands and the Cu(I) complexes in this study have been synthesized according to procedures reported in our previous work.^39^

### Solar Cell Fabrication

*Preparation of Working Electrodes*: Pilkington TEC15 substrates were sequentially cleaned in an ultrasonic bath first with a detergent solution (RBS 25 from Fluka Analytical), then deionized water, and finally ethanol. After drying in air, the substrates were screen-printed (active area, 0.36 cm^2^) with a transparent TiO_2_ paste (two layers, GreatCell Solar 18NR-T) and then dried at 125 °C for 6 min before being screen-printed with a TiO_2_ scattering layer paste (two layers, Solaronix, WER2-O). The samples were heated gradually at 180 °C (10 min), 320 °C (10 min), 390 °C (10 min), and 450 °C (60 min) in an oven (Nabertherm Controller P320) under ambient air atmosphere. After sintering, the samples were immersed into a 40 mM aqueous TiCl_4_ solution at 70 °C for 60 min. Subsequently, the substrates (total thickness around 12–14 μm) were removed from the solution, rinsed with deionized water, dried, and exposed to a final heating step (500 °C for 30 min). The TiO_2_ layer was sensitized with the copper(I) complexes according to the procedure reported in the “Self-Assembly of the Complexes on TiO_2_” section. *Preparation of Counter Electrodes*: A predrilled one-hole Pilkington TEC8 glass substrate was cleaned following the same procedure as reported for the working electrodes and heated in air at 400 °C for 30 min to remove residual impurities. After cooling to room temperature, 10 μL cm^–2^ of a 4.8 mM H_2_PtCl_6_ solution in ethanol was deposited on the glass substrate, followed by heating in air at 400 °C for 30 min. *Solar Cell Assembly*: The working electrode was sealed in a sandwich structure with the counter electrode using a 25 μm thick thermoplastic Surlyn frame (Meltonix 1170-25 from Solaronix). The electrolyte was introduced into the sealed devices through the predrilled hole by a vacuum backfilling technique. Finally, the hole in the counter electrode was sealed with a thermoplastic Surlyn film with a glass coverslip on top by heating at 120 °C for 20 s.

### Self-Assembly of the Complexes on TiO_2_

The TiO_2_ substrates were soaked in a 1.0 mM methanol solution of the ligand dbda for 24 h at room temperature. Each electrode was removed from the solution, washed with methanol, and dried with compressed air. Each functionalized electrode was thereafter soaked at room temperature for 24 h in a 1 mM acetonitrile solution of the desired homoleptic complexes [Cu(dmp)_2_]^+^, [Cu(Br-dmp)_2_]^+^, [Cu(bcp)_2_]^+^, [Cu(dsbtmp)_2_], and [Cu(dap)_2_]^+^, or, in an acetonitrile solution containing 1 mM [Cu(CH_3_CN)_4_]PF_6_ and 2 mM biq ligand, alternatively in a methanol solution containing 1 mM [Cu(CH_3_CN)_4_]PF_6_ and 2 mM of the dbda ligand. As a final step, the electrodes were removed from the dye-bath solution and washed with acetonitrile.

### Film Thickness Characterization

The thickness of the TiO_2_ electrode layer was determined by means of a profilometer (Veeco Dektak 150).

### Photovoltaic Device Characterization

Current–voltage (*I*–*V*) measurements were carried out with a Keithley 2400 source/meter and a Newport solar simulator (model 91160); the light intensity was calibrated using a certified reference solar cell (Fraunhofer ISE) to an intensity of 1000 W m^–2^ (AM 1.5G spectrum). The efficiencies reported have been recorded without using a mask. Incident photon-to-current conversion efficiency (IPCE) spectra were recorded by a computer-controlled setup comprising a xenon lamp (Spectral Products ASB-XE-175), a monochromator (Spectral Products CM110), and a Keithley multimeter (model 2700), calibrated by a certified reference solar cell (Fraunhofer ISE). For the IPCE spectra, a black mask with an aperture slightly smaller than the active area of the cell was applied on top of the cell (0.5 × 0.5 cm^2^).

### Density Functional Theory (DFT) Calculations

The copper(I) complexes were studied using density functional theory (DFT) calculations. All calculations were carried out using the program package Gaussian 16 (rev. B.01).^49^ The molecular structures were geometrically optimized using the cam-B3LYP hybrid functional.^50^ 6-311G basis sets were used for all light elements (H, C, N, O). Small-core, effective-core potentials (MDF10) in combination with a double-ζ-quality valence space were used for Cu and Br.^51,52^

### Hard X-ray Photoelectron Spectroscopy (HAXPES)

HAXPES measurements were carried out in the HIKE end station at the KMC-1 beamline of the BESSY II synchrotron facility at the Helmholtz-Zentrum Berlin.^53^ The available photon energy range at this beamline is 2–12 keV. A thin film of the [Cu(Br-dmp)_2_]^+^ homoleptic complex on a fluorine-doped tin oxide (FTO) substrate and the [Cu(dbda)(Br-dmp)]^+^ heteroleptic complex on TiO_2_ was studied. As references, a pristine TiO_2_ film and a TiO_2_ film with the anchoring ligand adsorbed to the surface were investigated. Experiments were carried out under ultrahigh vacuum conditions in the analysis chamber with a photon energy of 3 keV, selected using a Si(111) double-crystal monochromator. A high-resolution hemispheric electron analyzer (VG Scienta R4000) was used for the detection of ejected electrons. Overview spectra were recorded for each sample with a pass energy of 500 eV (see Figure S1, Supporting Information). Following this, high-resolution, core level spectra were obtained from a fresh sample spot with a pass energy of 200 eV and measurements from the same regions were repeated several times to monitor sample charging and potential X-ray beam damage. A shift in peak positions was observed after the first scan of core level spectra due to sample charging caused by the incident X-ray radiation (see the Supporting Information). For this reason, internal references of the core levels of interest obtained before and after measurements were used for binding energy calibration when comparing different samples: either the Ti 2p_3/2_ level of the TiO_2_ substrate was set to 458.6 eV or the adventitious C 1s peak was set to 284.8 eV.^54^ Both calibration methods yielded similar binding energies. The spectra were modeled by pseudo-Voigt functions to evaluate peak intensities used for normalization of the spectra.^55^ Where appropriate, elemental ratios were determined from the peak intensities using the photoionization cross section calculated by Scofield et al.^56^

## Results and Discussion

**Scheme 1:**
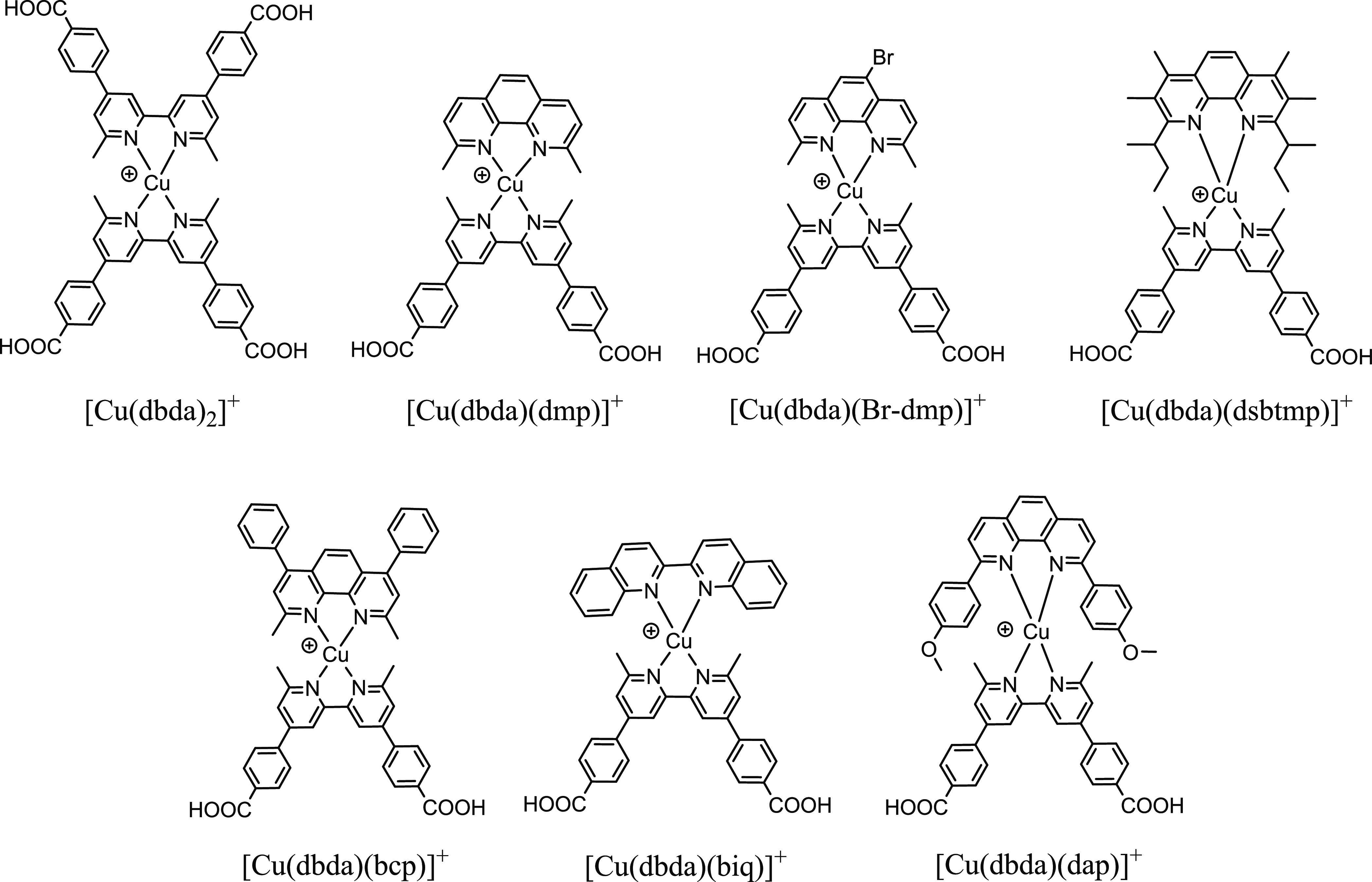
Molecular Structures of the Surface-Assembled Heteroleptic Cu(I) Complexes Investigated as Dyes for DSSCs in This Work

In our previous work,^39^ we followed the self-assembly method employed by Housecroft and Constable to prepare a series of Cu(I) complexes directly on the surface of the TiO_2_ substrate (Scheme 1).^47,48^

**Figure 1:**
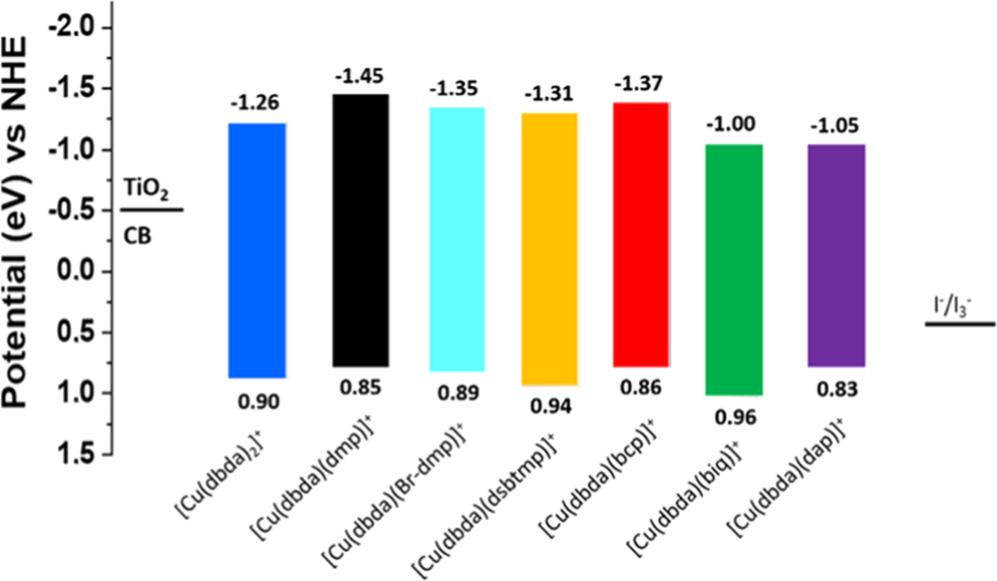
Energy level alignments of the Cu(I) complexes in Scheme 1 with respect to the CB edge of TiO_2_ and the potential of the redox couple I^–^/I_3_^–^. The values are reported as potential vs normal hydrogen electrode (NHE).

The optical and electrochemical properties of the complexes were investigated as well, and based on those results, it appeared evident that the complexes anchored on the surface of TiO_2_ showed a potential to be employed as dyes for DSSCs. In Figure 1, the energy levels of the copper(I) complexes derived from cyclic voltammetry and optical analysis^39^ are shown together with their alignment with respect to the CB edge of TiO_2_ and the redox potential of the redox couple I^–^/I_3_^–^ used in this study.

From a purely energetic perspective, the complex [Cu(dbda)(dmp)]^+^ shows the most negative lowest unoccupied molecular orbital (LUMO) energy (−1.45 eV vs NHE) within the series of complexes studied and thus the highest driving force for electron injection into the CB of TiO_2_. The complex [Cu(dbda)(biq)]^+^ instead shows the most positive highest occupied molecular orbital (HOMO) energy (0.96 eV vs NHE), which could favor the process of regeneration of the oxidized complex. This may be especially valid in the case of the I^–^/I_3_^–^ redox couple employed, as it is well known for requiring a significant driving force for efficient regeneration of the oxidized dye.^57^ However, we would like to point out that, given the labile nature of the Cu(I) complexes,^41^ the energetic alignments may play a limited role in the determination of the photovoltaic properties. Instead, the binding affinity of the ancillary ligand to the copper center, as well as its ability to efficiently screen the copper center from solvent interactions, may play the most crucial role in the device performance.

**Figure 2:**
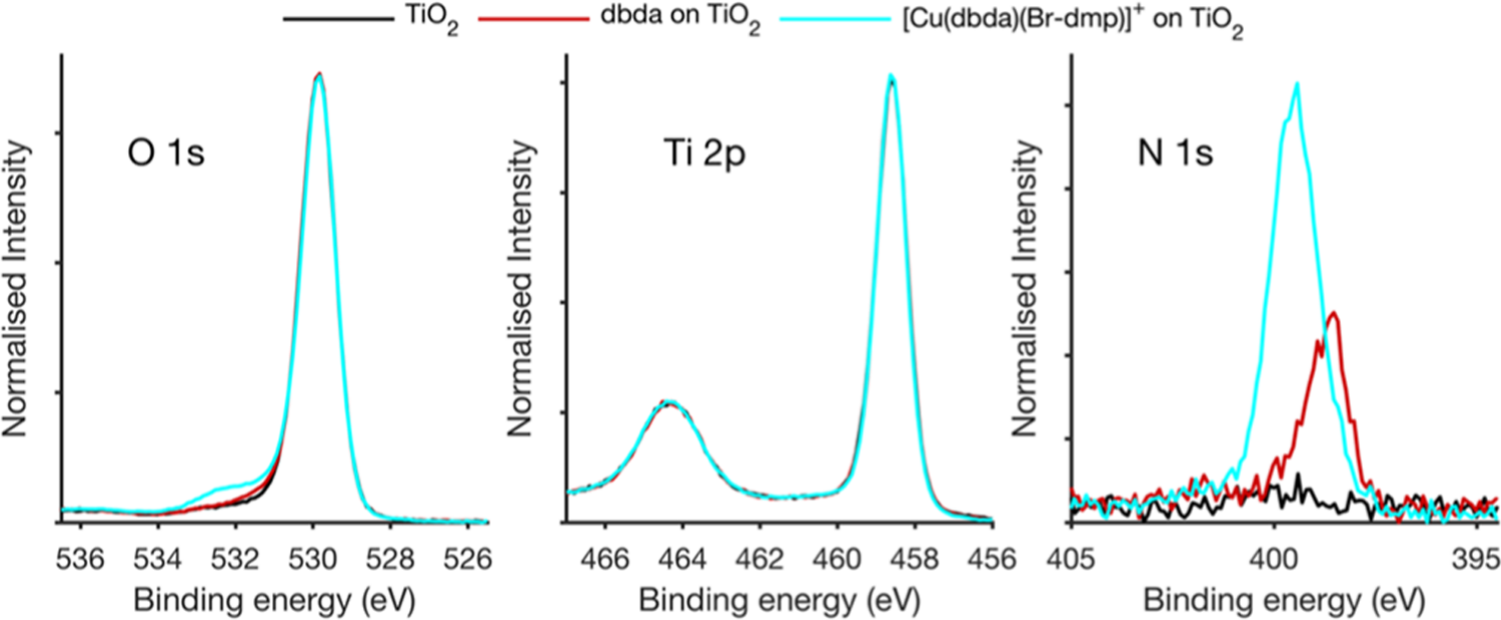
Photoelectron spectra of [Cu(dbda)(Br-dmp)]^+^ adsorbed on TiO_2_ as compared to the TiO_2_ substrate and the anchoring ligand on the substrate studied at a photon energy of 3000 eV. The energy is calibrated to the Ti 2p_3/2_ peak at 458.6 eV. The intensity is normalized to the Ti 2p_3/2_ peak intensity.

We have investigated the formation of the heteroleptic complexes on the TiO_2_ surface by hard X-ray photoelectron spectroscopy using the [Cu(dbda)(Br-dmp)]^+^ complex as an example. The addition of Br to the ligand of the homoleptic complex helps us to distinguish the two ligands on the surface and therefore to follow the assembly onto the TiO_2_ surface. Figure 2 shows the core level spectra of the heteroleptic complex compared to those of the anchoring ligand adsorbed on TiO_2_ and of a pristine TiO_2_ surface normalized to the intensity of the Ti 2p_3/2_ peak determined for each sample. The main peaks for the O 1s spectra (530 eV) clearly overlap for the three samples and correspond to the oxygen atoms in TiO_2_. A small side shoulder at higher binding energies is observed for all samples, which could originate from molecules adsorbed to the surface and/or surface contamination. This shoulder is significantly more intense for the assembled copper complexes (discussed below). The N 1s spectra show a clear difference, confirming the formation of Cu complexes on the surface. For the sample based on TiO_2_ alone, almost no signal from nitrogen is observed, while a clear peak from nitrogen is observed after binding the dbda ligand to the TiO_2_ surface. Upon formation of the Cu complex, the nitrogen peak increases in the intensity and its position shifts relative to the substrate reference peaks. This reveals that the formation of the Cu complex leads to additional nitrogen on the surface, as well as to a change of the chemical environment of nitrogen in the dbda ligand. The nitrogen peak for [Cu(dbda)(Br-dmp)]^+^ is shifted to higher binding energies, in agreement with the presumed additional bond formation between copper and the nitrogen atoms in the dbda ligand. This suggests that the [Cu(dbda)(Br-dmp)]^+^ complex forms efficiently on the surface and that little or no unbound dbda ligand remains.

**Figure 3:**
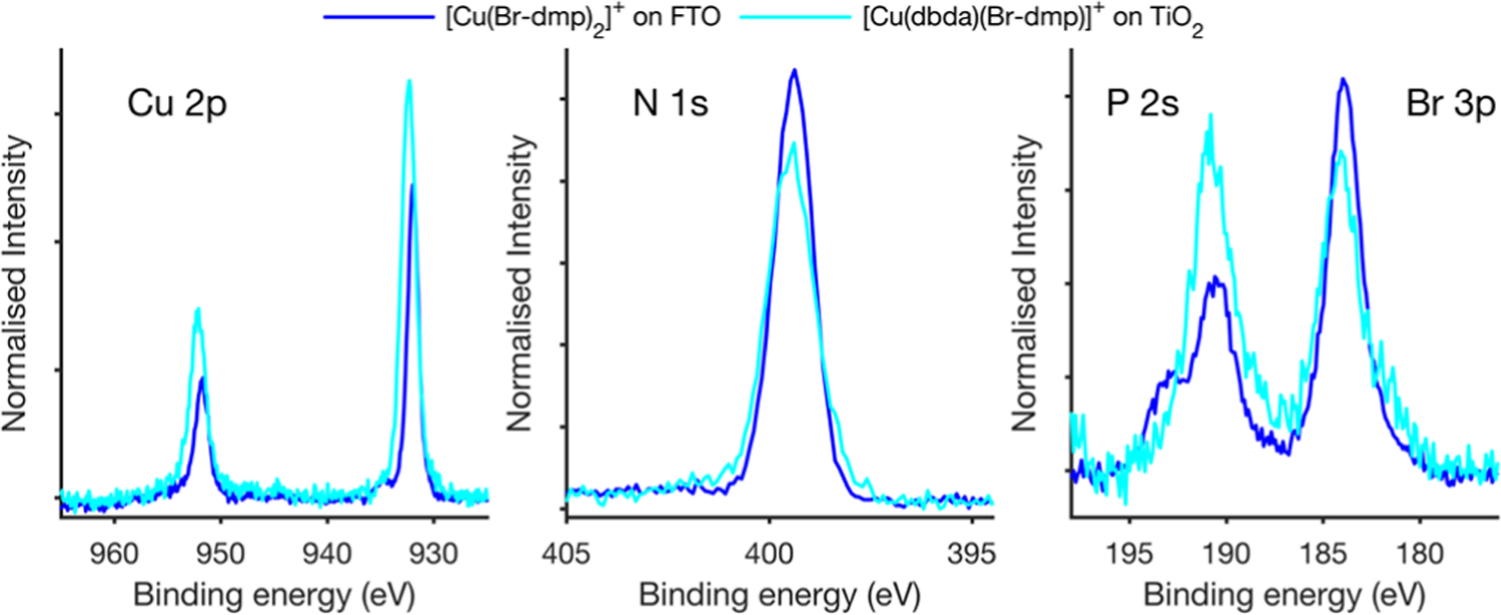
Photoelectron spectra of [Cu(Br-dmp)_2_]^+^ and [Cu(dbda)(Br-dmp)]^+^ adsorbed on TiO_2_ determined at a photon energy of 3000 eV. The energy is calibrated to the C 1s peak at 284.8 eV. The intensity is normalized to the N 1s peak intensity.

**Figure 4:**
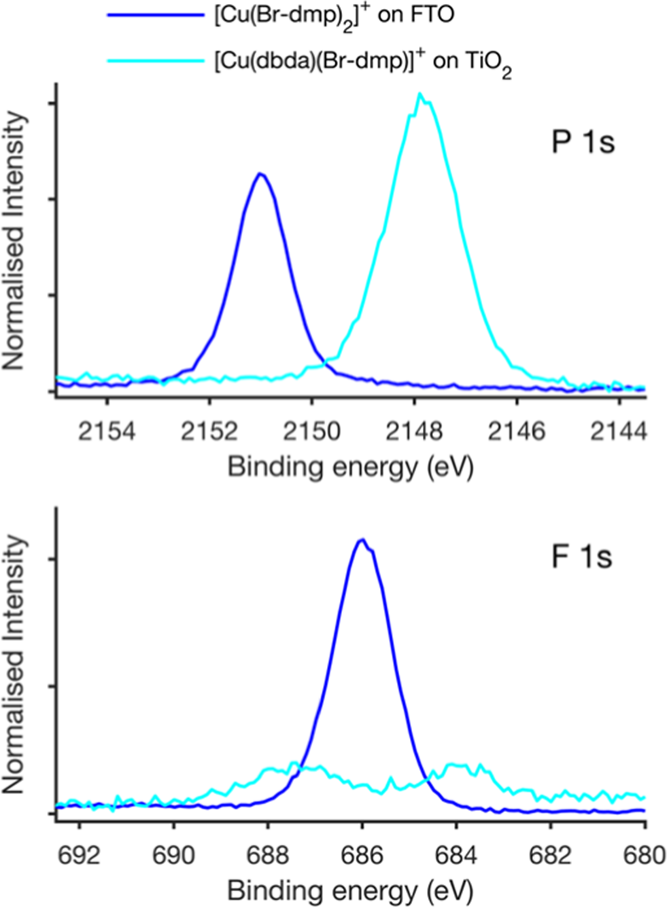
Photoelectron spectra of the counterion (PF_6_^–^) core levels of [Cu(Br-dmp)_2_]^+^ and [Cu(dbda)(Br-dmp)]^+^ adsorbed on FTO and TiO_2_ obtained for a photon energy of 3000 eV. The energy is calibrated to the C 1s peak at 284.8 eV. The intensity is normalized to the N 1s peak intensity.

To further investigate the stoichiometry and formal oxidation state of the heteroleptic complexes, we compared [Cu(dbda)(Br-dmp)]^+^ adsorbed on TiO_2_ to its homoleptic equivalent ([Cu(Br-dmp)_2_]^+^) deposited on a FTO substrate. The thin film of the [Cu(Br-dmp)_2_]^+^ homoleptic complex deposited on FTO only showed a weak signal from the substrate present. To compare peak positions and intensities, the spectra were energy calibrated against the adventitious C 1s peak at 284.8 eV and normalized to the N 1s peak intensity obtained through fitting to a Voigt function. Figure 3 shows the core level spectra relating to the complex itself (Cu 2p, N 1s, and Br 3p), while Figure 4 shows the core level spectra relating to the PF_6_^–^ counterion (P 1s and F 1s).

The N 1s peak is found at a similar position in both samples but is wider for the heteroleptic complex when adsorbed to TiO_2_. The Cu 2p core level reveals the presence of some Cu(II) for the homoleptic complex based on the presence of an extra intensity around 940 eV and a satellite feature at 936 eV (Figure S2). However, during X-ray irradiation, Cu(II) was quickly converted to Cu(I) and quantification of Cu(II) was therefore not possible. For the heteroleptic complex adsorbed to TiO_2_, only Cu(I) was observed, and the constant peak feature indicates that no change takes place during X-ray radiation (Figure S3). The comparison of the spectra of [Cu(Br-dmp)_2_]^+^ and [Cu(dbda)(Br-dmp)]^+^ reveals that the Cu 2p peak is wider and more intense in relation to the N 1s peak for the heteroleptic Cu complex adsorbed to TiO_2_. This is indicative of a difference in the number of nitrogen atoms per copper atom for the complex when adsorbed to the TiO_2_ surface in comparison with the homoleptic complex. This observation could have two nonexclusive explanations: (1) an excess of unbound ligands in the sample of the homoleptic compound and/or (2) incomplete formation of the heteroleptic complexes on the TiO_2_ surface. In the latter case, Cu(I) would bind to the dbda ligand on the surface but, to some extent, lack the Br-dmp ligand or would be deposited on the surface without coordinating ligands. Since we only observe one narrow nitrogen peak, it is unlikely that there is a significant excess of ligands in the homoleptic sample. Quantification of the N to Cu ratio based on photoionization cross sections described by Scofield et al.^56^ gives a N/Cu ratio between 4.5 and 4 to 1 (Table S1). However, due to the difference in molecular orbital composition and energies (s and p), differences in kinetic energies, uncertainties in the cross section, and variations in the Cu 2p spectra, this quantification should be taken with some reservation.

A further indication of the ligands present in the heteroleptic complexes can be obtained from the Br signal intensity using the feature of the Br 3p_3/2_ peak (∼183 eV). This peak was chosen due to overlapping peaks for several other orbitals as explained in the Supporting Information (Figure S10). The spectra from the heteroleptic complex were multiplied by a factor of 2 to compensate for the difference in the stoichiometric ratios of N/Br in the homoleptic complex (2:1) and heteroleptic complex (4:1). If half of the ligands of the heteroleptic complex are Br-dmp, we would expect the same Br peak intensities in the Br 3p spectrum in Figure 3 as from the homoleptic compound. While the Br 3p intensity for the heteroleptic complex looks somewhat lower than that from the homoleptic complex, this is not confirmed by quantification based on modeling the spectra (Tables S1 and S2). The total Br/N ratio for [Cu(Br-dmp)_2_]^+^ is 0.5 and for [Cu(dbda)(Br-dmp)]^+^, it is 0.25, both as expected from the formal stoichiometry. This observation suggests an equal number of dbda and Br-dmp ligands in the complex adsorbed on the TiO_2_ surface. Combined with the differences in Cu peak intensities, it follows that some copper adsorbed to the surface is uncoordinated to either Br-dmp or dbda ligands.

The counterion of the homoleptic complex is PF_6_^–^ and peaks from this species are observed in the P 1s and F 1s spectra (Figure 4). Quantification suggests an F/P ratio between 6 and 7 to 1. When absorbed to the TiO_2_ surface, a strong P 1s peak is observed, but it is shifted to lower binding energies by about 3 eV. The F 1s signal intensity decreases significantly, and two peaks are observed instead of one when adsorbed on the metal-oxide surface. This indicates that the PF_6_^–^ counterion is therefore not present on the TiO_2_ surface. A possible mechanism for the loss of fluorine would be through a reaction with protons from the TiO_2_ surface generating HF, which could escape to the solution leaving phosphorous behind in a chemically different state. Furthermore, for this particular sample, another phosphorous-containing species seems to be present on the surface. The shift to lower binding energies is in agreement with the formation of oxide-containing phosphates.^58^ An O 1s signal from such phosphates is expected at higher binding energies than O 1s from the metal oxides.^59^ For the [Cu(dbda)(Br-dmp)]^+^ complex, a higher O 1s peak intensity is observed to the high binding energy side of the TiO_2_ O 1s peak as compared to the references (Figure 3), consistent with oxide-based phosphates. This, therefore, suggests that the PF_6_^–^ counterion is converted to phosphate on the TiO_2_ surface.

**Figure 5:**
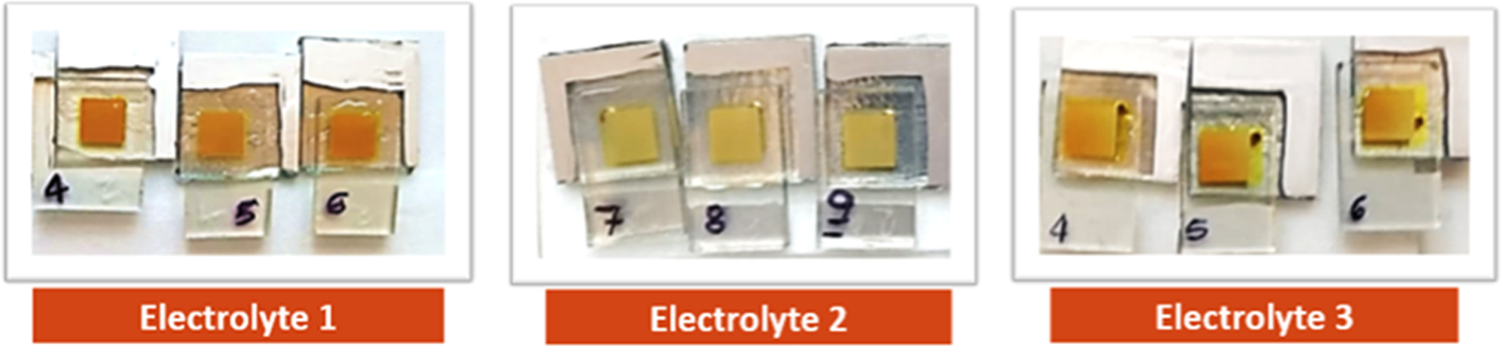
Complete DSSCs based on the dye [Cu(dbda)(bcp)]^+^ in which different iodide/triiodide electrolyte compositions have been injected. Electrolyte 1: 0.65 M 1-butyl-3-methylimidazolinium iodide, 0.025 M LiI, 0.04 I_2_, 0.28 M 4-*tert*-butylpyridine (TBP) in acetonitrile/valeronitrile (volume ratio: 85/15);^60^ electrolyte 2: 1.0 M 1-butyl-3-methylimidazolinium iodide, 0.1 M LiI, 0.05 I_2_, 0.5 M TBP in acetonitrile; electrolyte 3: same composition as for electrolyte 2 but in acetonitrile/valeronitrile (volume ratio: 85/15).

To identify the optimal conditions for DSSC fabrication, we used the complex [Cu(dbda)(bcp)]^+^ as a test dye. While we report a part of the optimization work in the Supporting Information (Tables S3 and S4), we would like to highlight some of our findings as they reveal interesting aspects regarding the behavior and properties of these Cu(I) complexes. In a first attempt, we tried to compare the efficiency of DSSCs based on the complex [Cu(dbda)(bcp)]^+^ using two different electrolyte redox couples: I^–^/I_3_^–^ and [Co(bpy)_3_]^2+/3+^ (Table S3). However, the electrolyte based on the cobalt redox system partly bleached the dyed TiO_2_ directly after injection, rendering DSSCs with lower performances. Given the instantaneous bleaching effect and the difficulties in formulating other Co-based electrolytes, we decided to use I^–^/I_3_^–^ as the redox mediator system for the rest of our study. Interestingly, when attempting to optimize the electrolyte composition, we again observed a bleaching phenomenon shown in Figure 5. The detailed photovoltaic parameters of the DSSCs based on the electrolytes 1 and 3 (Figure 5) are shown in Table 1.

**Table 1:** Photovoltaic Details of DSSCs Based on the Electrolytes 1 and 3 shown in Figure 5^a^

electrolyte	η (%)	*V*_OC_ (mV)	*J*_SC_ (mA cm^–2^)	FF (%)
1	2.07 ± 0.09	622 ± 5	4.693 ± 0.10	71 ± 1
3	1.74 ± 0.10	577 ± 5	4.192 ± 0.11	72 ± 1

a The average values reported are based on three devices 2 days after sealing. The efficiencies of the devices based on electrolyte 2 are extremely low (≈0.005%) and the photovoltaic parameters are therefore not included in the table.

**Figure 6:**
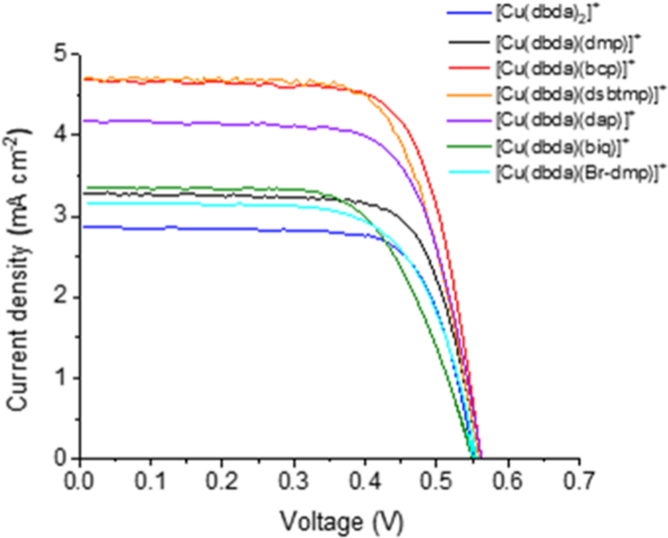
Current–density characteristics of DSSCs based on the Cu(I) dyes shown in Scheme 1, under AM 1.5G, 1 sun illumination using the electrolyte composition 1 (Figure 5). The results refer to an average of three solar cells recorded two days after assembly.

We do not expect the anchoring ligand dbda to desorb from the TiO_2_ surface under the conditions employed, but we are aware of the lability of the Cu(I) complexes in terms of ligand scrambling.^61^ Therefore, a reasonable conclusion is that one or more components in the electrolytes may in part exchange with the ancillary ligand that is coordinated to the metal center. From the electrolyte compositions investigated, we can make the following conclusions: (1) the use of pure acetonitrile as an electrolyte solvent causes significant bleaching regardless of the redox couple used (see electrolyte 2, Figure 5); (2) high concentrations of TBP and of all of the electrolyte components cause partial bleaching (see electrolytes 2 and 3, Figure 5); and (3) 3-methoxypropionitrile causes significant bleaching (Table S4). This is in agreement with a previous report, where 6,6′-dimethyl substituted 2,2′-bipyridine anchoring ligand provide heteroleptic copper complexes stable in electrolytes when TBP is not used and 3-methoxypropionitrile is used as a solvent instead of acetonitrile.^36^ Therefore, we found that electrolyte 1 composition reported by Colombo et al.^60^ offered DSSCs with the best photovoltaic performance. The current–density characteristics of DSSCs assembled with N719 and the copper(I) dyes in this study are shown in Figure 6, and the detailed photovoltaic parameters are reported in Table 2. The lower efficiency of the Cu-based solar cells with respect to the N719-based one was ascribed to lower light harvesting, in agreement with lower extinction coefficients (∼7500 and ∼15 000 M^–1^ cm^–1^ for Cu complexes and N719, respectively) and less broad absorption spectra (350–650 and 350–800 nm for Cu complexes and N719, respectively) as previously reported.^39^

**Table 2:** Detailed Photovoltaic Parameters of the *J*–*V* Characteristics Shown in Figure 6 and Reference DSSCs Assembled Using the Dye N719^a^

dye	η (%)	*V*_OC_ (mV)	*J*_SC_ (mA cm^–2^)	FF (%)
[Cu(dbda)_2_]^+^	1.17 ± 0.09	550 ± 10	2.87 ± 0.10	74 ± 2
[Cu(dbda)(dmp)]^+^	1.38 ± 0.10	563 ± 5	3.31 ± 0.12	74 ± 2
[Cu(dbda)(Br-dmp)] ^+^	1.23 ± 0.10	555 ± 5	3.17 ± 0.11	70 ± 1
[Cu(dbda)(dsbtmp)]^+^	1.81 ± 0.12	563 ± 5	4.79 ± 0.11	68 ± 1
[Cu(dbda)(bcp)]^+^	2.05 ± 0.08	565 ± 10	4.79 ± 0.07	73 ± 1
[Cu(dbda)(biq)]^+^	1.24 ± 0.09	553 ± 5	3.35 ± 0.09	67 ± 2
[Cu(dbda)(dap)]^+^	1.73 ± 0.09	566 ± 5	4.16 ± 0.10	72 ± 1
N719	7.60 ± 0.21	700 ± 5	17.81 ± 0.09	61 ± 1

a The values originate from three DSSCs of each type investigated 2 days after assembly. Electrolyte 1 (Figure 5) was used.

**Figure 7:**
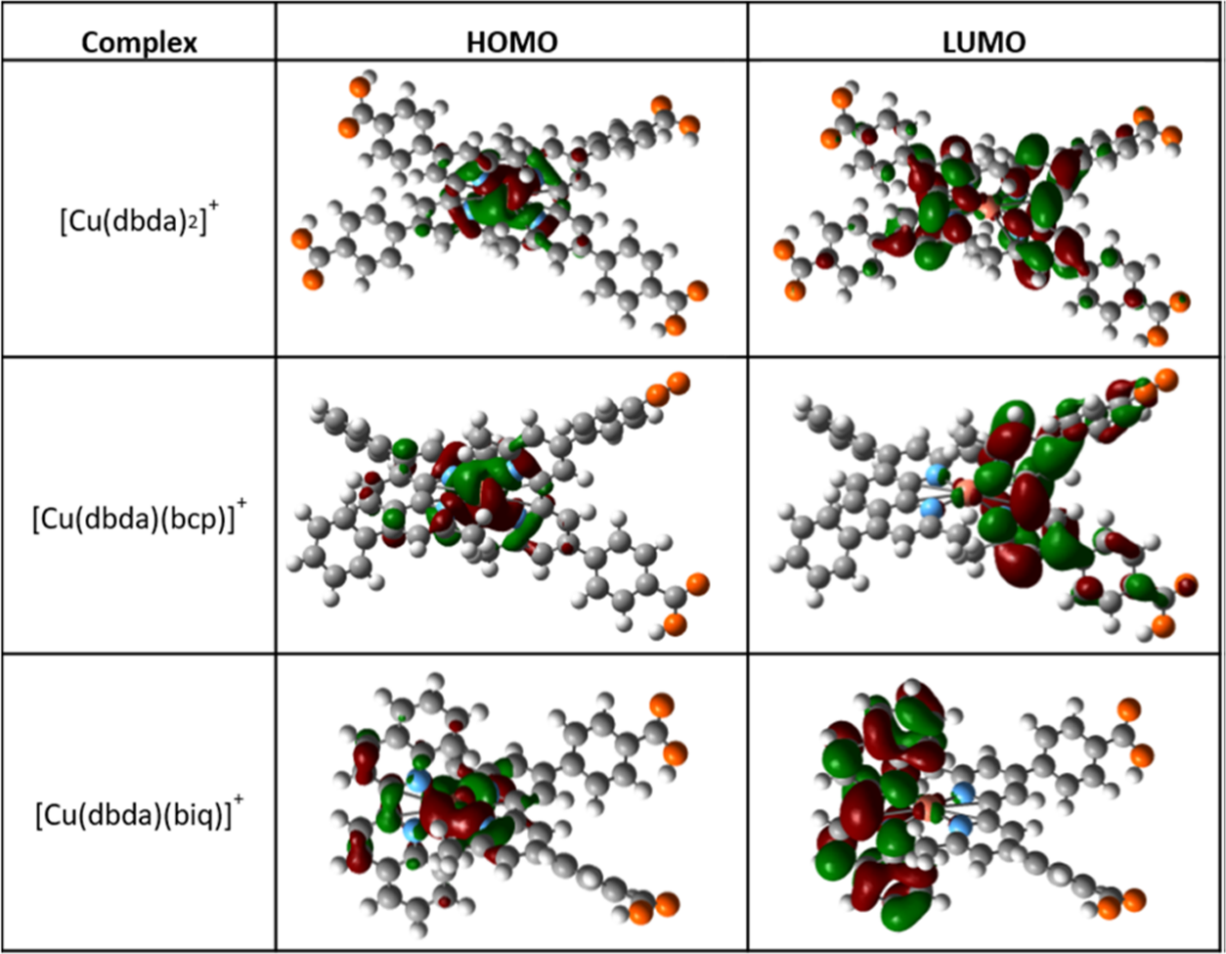
Calculated HOMO and LUMO distribution of the copper(I) complexes. Hydrogen atoms are shown in white, carbon atoms in gray, nitrogen atoms in light blue, and oxygen atoms in orange. The calculated ground-state geometries, as well as HOMO and LUMO electronic distribution, for all of the complexes are reported in Figure S11.

Going from the lowest to the highest efficiency of the DSSCs based on the series of the Cu(I) complex, we find the following trend: [Cu(dbda)_2_]^+^, [Cu(dbda)(Br-dmp)]^+^ ≈ [Cu(dbda)(biq)]^+^, [Cu(dbda)(dmp)]^+^, [Cu(dbda)(dap)]^+^, [Cu(dbda)(dsbtmp)]^+^, and [Cu(dbda)(bcp)]^+^. To get better insights into the performances of the dyes, the HOMO and LUMO energy level distributions in the complexes were calculated using DFT (Figure 7).

In analogy to the ruthenium dyes, the HOMO electron density distribution is also located around the metal coordination center in all of the studied copper complexes.^62,63^ The complex [Cu(dbda)_2_]^+^ has previously been investigated by Melchiorre et al.,^60^ and they used the complex for the fabrication of DSSCs that showed 3.0% power conversion efficiency (η). It is interesting to point out that, despite the difference in absolute efficiency between our device and their DSSC devices, all of the other complexes investigated in this work performed better than [Cu(dbda)_2_]^+^. Compared to the heteroleptic complexes, the worst performance of the DSSCs based on the complex [Cu(dbda)_2_]^+^ is mostly related to its lower photocurrent (*J*_sc_). Being a homoleptic complex, the [Cu(dbda)_2_]^+^ structure does not promote a metal-to-ligand charge transfer (MLCT) selectively to the ligand anchored on the surface of TiO_2_. Instead, since the LUMO is equally distributed on both ligands (Figure 7), a reasonable assumption would be that the probability that the MLCT leads to an electron promoted to the anchoring ligand is ≈50%. Therefore, as a result, the process of electron injection into the semiconductor CB is expected to be statistically less likely, causing the lower *J*_sc_ and open-circuit voltage (*V*_oc_) observed. The DSSCs based on the complexes [Cu(dbda)(Br-dmp)]^+^ and [Cu(dbda)(biq)]^+^ show very similar efficiency and *V*_oc_ (Table 2). Despite the LUMO being fully delocalized on the biq ligand (Figure 7), the complex [Cu(dbda)(biq)]^+^ yields a slightly higher photocurrent, however, counteracted by the lower fill factor (FF), suggesting a higher degree of carrier recombination loss. The devices based on the complex [Cu(dbda)(dmp)]^+^ as dyes perform slightly better as compared to those based on the brominated analog [Cu(dbda)(Br-dmp)]^+^. Both the complexes [Cu(dbda)(Br-dmp)]^+^ and [Cu(dbda)(dmp)]^+^ display LUMOs delocalized on the anchoring dbda ligand (Figure S11), and therefore, the difference in the solar cell performance cannot be simply explained in terms on differences in spatial HOMO/LUMO distributions. The sensitizer [Cu(dbda)(dap)]^+^ yields a good performance within the series, which is most likely due to the high *J*_sc_. From a chemical perspective, the ligand dap contains bulky-donating anisyl substituents in the 2,9 positions of the 1,10-phenanthroline core. As a result, the structure of this heteroleptic Cu(I) complex strongly resembles the push–pull design commonly employed for organic sensitizers.^64−67^ In this case, the donating dap ligand, being electron-rich, actively “pushes” the MLCT toward the anchoring dbda ligand and, in turn, into the TiO_2_ CB resulting in a higher *J*_sc_. DSSCs based on the sensitizer [Cu(dbda)(dsbtmp)]^+^ showed the highest photocurrent within the series. This is quite interesting as the result cannot be trivially attributed to the different light absorption properties (Figure S12) or to the donating/withdrawing character of the ancillary ligand of the complex. The ligand dsbtmp was reported by McCusker and Castellano,^68^ and despite showing a rather low molar extinction coefficient, its resulting homoleptic Cu(I) complex exhibited relatively high stability attributed to the ability of the *sec*-butyl groups to efficiently screen the metal center from solvent coordination, thus retarding detrimental ligand exchange reactions. Therefore, we speculate that the affinity constant of this ligand is higher than that for the other ligands, leading to a higher effective amount of this complex being assembled on the surface of TiO_2_, in turn resulting in a higher *J*_sc_. Finally, devices based on the dye [Cu(dbda)(bcp)]^+^ offer photocurrent very similar to those based on the dye [Cu(dbda)(dsbtmp)]^+^, but the slightly higher efficiency recorded for the resulting DSSCs originates from a higher FF. The ligand bcp, like dap, is electron donating in character, thus able to push the MLCT toward the TiO_2_ surface as shown by its LUMO being delocalized on the dbda ligand (Figure 7). Moreover, the relatively high molar extinction coefficients of both the homoleptic complexes [Cu(dbda)_2_]^+^ and [Cu(bcp)_2_]^+ ^^39,60,69^ suggest that there is also a chance that the heteroleptic complex [Cu(dbda)(bcp)]^+^ may be a good light absorber. The reference N719 system (Table 2) shows a superior performance, which is determined by the significantly higher photocurrent around four times higher than that of the DSSCs based on the best Cu(I) complex in this study and can in part be attributed to the broader light absorption by N719 than from any of the Cu(I) complexes.

**Figure 8:**
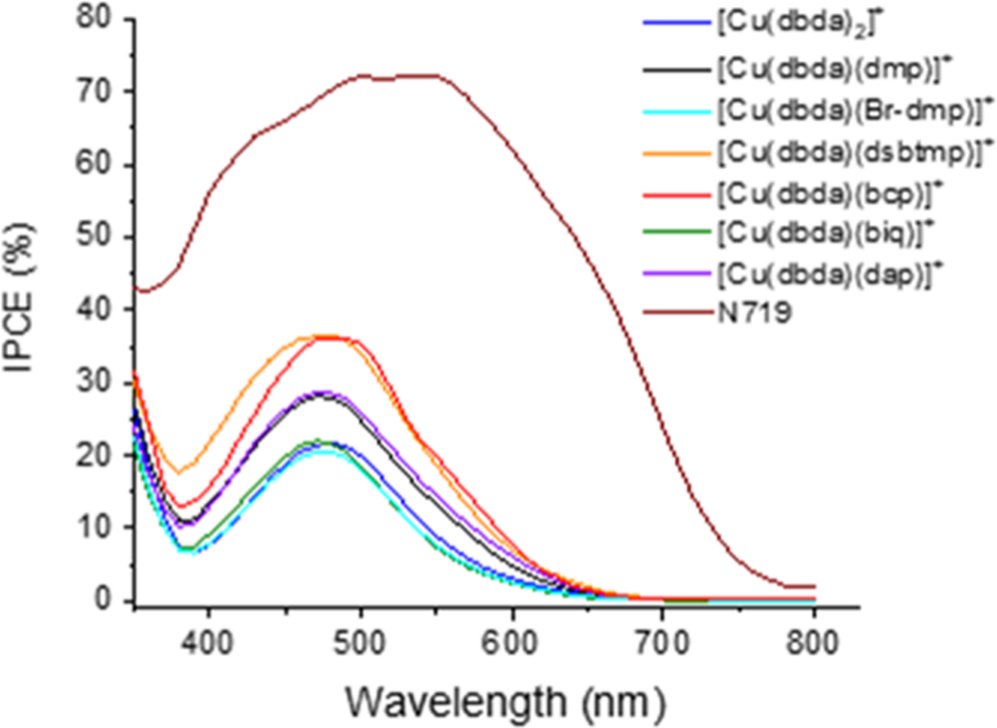
IPCE spectra of the DSSCs sensitized by Cu(I) complexes in this study and the reference N719 dye. Each curve represents the average of 3 DSSCs. The investigation is performed on the device 2 days after assembly.

The IPCE spectra of the DSSC devices based on the Cu(I) complexes and the ruthenium dye N719 are shown in Figure 8. Within the series, from the highest to the lowest IPCE maxima, we find the order [Cu(dbda)(dsbtmp)]^+^ followed by [Cu(dbda)(bcp)]^+^, [Cu(dbda)(dap)]^+^, [Cu(dbda)(dmp)]^+^, [Cu(dbda)(biq)]^+^, [Cu(dbda)_2_]^+^, and [Cu(dbda)(Br-dmp)]^+^. As expected, this trend is coherent with the current densities obtained for the respective devices. Table 3 shows a comparison between the wavelengths associated with the absorption maxima of the dyes adsorbed on TiO_2_ (Figure S12) and the maximum intensity of the IPCE.

**Table 3:** Maximum Absorption Wavelengths of the Cu(I) Dyes Adsorbed on TiO_2_ (Figure S12) and Wavelengths of the IPCE Maxima Reported in Figure 8^a^

dye	**λ**_**max**_ (nm)^a^	λ_IPCEmax_ (nm)
[Cu(dbda)_2_]^+^	474	480
[Cu(dbda)(dmp)]^+^	459	474
[Cu(dbda)(Br-dmp)]^+^	451	473
[Cu(dbda)(dsbtmp)]^+^	460	473
[Cu(dbda)(bcp)]^+^	471	485
[Cu(dbda)(biq)]^+^	531	473
[Cu(dbda)(dap)]^+^		473

a Figure S12.

Curiously, except for the benchmark N719 and despite the diverse intensities, most of the IPCE spectra show the same shape and λ_IPCEmax_ (473–474 nm). The sole exceptions to the general pattern are represented by the IPCE spectra related to the complexes [Cu(dbda)(bcp)]^+^ and [Cu(dbda)_2_]^+^, which display a slightly more red-shifted λ_IPCEmax_ as compared to the rest of the series (485 and 480 nm, respectively). Of particular interest is the difference between the maximum absorption and IPCE wavelengths related to the complex [Cu(dbda)(biq)]^+^, which is around 60 nm. As shown from Figure 7, a possible explanation is that the LUMO for the [Cu(dbda)(biq)]^+^ complex is located on the biq ligand, which is not likely to contribute to generating photocurrent. Also, it is surprising how a great change in the appearance of the sensitized TiO_2_ before and after electrolyte injection makes the sample [Cu(dbda)(biq)]^+^ look like all of the other samples (such evidence is further discussed in the Supporting Information after Figure S13). The intensity and the shape of the IPCE spectra hint at the cause for the observed performance of the DSSCs in this study. In particular, the shape of all of the Cu(I) complex IPCE spectra is characterized by a well-defined broad band as observed for the light absorption spectra of most of the complexes adsorbed on TiO_2_. This observation, combined with the relatively low intensity of the IPCE spectra and the high FF of the DSSCs (Table 2), suggests that the reason for the low overall performance may be related to low light-harvesting ability of the dyes themselves. As indicated from the calculations of the HOMO and LUMO energy levels (Figures 7 and S11), for the majority of the molecules, the electron density in the ground state is located on Cu(I), whereas for the LUMO, it is primarily positioned on the anchoring ligand dbda. The anchoring ligand dbda appears to dominate the electronic properties for the molecules and the ancillary ligands impose a more limited effect on photovoltaic performance by affecting the donation of electron density to the Cu(I) center. Finally, consistent with the results reported in Table 2, the IPCE spectrum related to the devices based on the dye N719 is significantly higher than those of the Cu(I) complexes.

## Conclusions

A series of Cu(I) diimine complexes were self-assembled on a TiO_2_ surface to investigate their efficacy as dyes in DSSCs. We closely studied the self-assembly of one of the complexes ([Cu(dbda)(Br-dmp)]^+^) on TiO_2_ by HAXPES. Our results suggest that the complex forms efficiently on TiO_2_ and that no significant amount of unbound dbda remains. However, some additional copper of unknown character not coordinated to either ligand might be deposited on the surface. In addition, the PF_6_^–^ counterion is not stable on the TiO_2_ surface and is either replaced by OH^–^ groups from the TiO_2_ surface or by phosphate ions formed through oxidation. The dyes were investigated in DSSCs, and their performance has been rationalized on the basis of the nature of their ancillary ligand. We have shown by theoretical calculations, absorption properties, and DSSC performances that the push–pull design is a key aspect for designing heteroleptic Cu(I) dyes with better performance: complexes including donating ancillary ligands display higher photocurrents that most likely can be attributed to a more efficient electron injection. Another important factor to consider is, not surprisingly, the molar extinction coefficients and spectral width of light absorption from the complexes. The results from the complex [Cu(dbda)(bcp)]^+^ suggest that, when designing a heteroleptic complex, it is important to use ligands for which the corresponding homoleptic Cu(I) complexes show high molar extinction coefficients.^39^ The dyes [Cu(dbda)(dsbtmp)]^+^ and [Cu(dbda)(dap)]^+^ contain the ancillary ligands dsbtmp and dap, respectively, whose corresponding homoleptic Cu(I) complexes display blue-shifted absorption spectra and low molar extinction coefficients. However, the heteroleptic complexes show very similar device performance as the best performing dyes in the series [Cu(dbda)(bcp)]^+^. This clearly highlights that there are factors, other than the optical properties, affecting the capacity of the complexes to inject electrons into the semiconductor. The inclusion of ancillary ligands with bulky substituents in the 2,9 positions of the phenanthroline core may screen the Cu(I) coordination center and bind more strongly to it. This may constitute another critical aspect of good solar cell performance. The low performance of DSSCs based on the complexes [Cu(dbda)_2_]^+^, [Cu(dbda)(Br-dmp)]^+^, and [Cu(dbda)(biq)]^+^ have, respectively, shown that homoleptic complexes, ancillary ligands containing electron-withdrawing substituents, and ancillary ligands with low complex formation constants should be avoided. In particular, the complex [Cu(dbda)(biq)]^+^ has revealed a dye–electrolyte interaction that, to various degrees, may also be present for the other Cu(I) dyes, a phenomenon that deserves to be further investigated.

## Supporting Information Available

XPS spectra; detailed photovoltaic parameters of nonoptimized DSSCs with different electrolyte compositions; DFT-calculated ground-state geometries of the dyes; absorption spectra of the dyes on TiO_2_; and N719 DSSC lifetime (PDF)

## References

[ref1] Grätzel M.; Kalyanasundaram K. Artificial Photosynthesis: Efficient Dye-Sensitized Photoelectrochemical Cells for Direct Conversion of Visible Light to Electricity. Curr. Sci. 1994, 66, 706–714.

[ref2] O’Regan B.; Grätzel M. A Low-Cost, High-Efficiency Solar Cell Based on Dye-Sensitized Colloidal TiO2 Films. Nature 1991, 353, 737–740. 10.1038/353737a0.

[ref3] Freitag M.; Teuscher J.; Saygili Y.; Zhang X.; Giordano F.; Liska P.; Hua J.; Zakeeruddin S. M.; Moser J. E.; Grätzel M.; Hagfeldt A. Dye-Sensitized Solar Cells for Efficient Power Generation under Ambient Lighting. Nat. Photonics 2017, 11, 372–378. 10.1038/nphoton.2017.60.

[ref4] Kawata K.; Tamaki K.; Kawaraya M. Dye-Sensitised and Perovskite Solar Cells as Indoor Energy Harvestors. J. Photopolym. Sci. Technol. 2015, 28, 415–417. 10.2494/photopolymer.28.415.

[ref5] Ren Y.; Sun D.; Cao Y.; Tsao H. N.; Yuan Y.; Zakeeruddin S. M.; Wang P.; Grätzel M. A Stable Blue Photosensitizer for Color Palette of Dye-Sensitized Solar Cells Reaching 12.6% Efficiency. J. Am. Chem. Soc. 2018, 140, 2405–2408. 10.1021/jacs.7b12348. 29323883

[ref6] Leandri V.; Ruffo R.; Trifiletti V.; Abbotto A. Asymmetric Tribranched Dyes: An Intramolecular Cosensitization Approach for Dye-Sensitized Solar Cells. Eur. J. Org. Chem. 2013, 2013, 6793–6801. 10.1002/ejoc.201300962.

[ref7] Leandri V.; Ellis H.; Gabrielsson E.; Sun L.; Boschloo G.; Hagfeldt A. An Organic Hydrophilic Dye for Water-Based Dye-Sensitized Solar Cells. Phys. Chem. Chem. Phys. 2014, 16, 19964–19971. 10.1039/c4cp02774d. 25119775

[ref8] Franchi D.; Calamante M.; Coppola C.; Mordini A.; Reginato G.; Sinicropi A.; Zani L. Synthesis and Characterization of New Organic Dyes Containing the Indigo Core. Molecules 2020, 25, 3377 10.3390/molecules25153377. PMC7435895 32722406

[ref9] Franchi D.; Calamante M.; Reginato G.; Zani L.; Peruzzini M.; Taddei M.; Fabrizi De Biani F.; Basosi R.; Sinicropi A.; Colonna D.; Di Carlo A.; Mordini A. Two New Dyes with Carboxypyridinium Regioisomers as Anchoring Groups for Dye-Sensitized Solar Cells. Synlett 2015, 26, 2389–2394. 10.1055/s-0035-1560713.

[ref10] Dessì A.; Sinicropi A.; Mohammadpourasl S.; Basosi R.; Taddei M.; Fabrizi de Biani F.; Calamante M.; Zani L.; Mordini A.; Bracq P.; Franchi D.; Reginato G. New Blue Donor–Acceptor Pechmann Dyes: Synthesis, Spectroscopic, Electrochemical, and Computational Studies. ACS Omega 2019, 4, 7614–7627. 10.1021/acsomega.8b03560. 31459854 PMC6648098

[ref11] Błaszczyk A. Strategies to Improve the Performance of Metal-Free Dye-Sensitized Solar Cells. Dyes Pigm. 2018, 149, 707–718. 10.1016/j.dyepig.2017.11.045.

[ref12] Carella A.; Borbone F.; Centore R. Research Progress on Photosensitizers for DSSC. Front. Chem. 2018, 6, 481 10.3389/fchem.2018.00481. 30364239 PMC6193062

[ref13] Aghazada S.; Nazeeruddin M. Ruthenium Complexes as Sensitizers in Dye-Sensitized Solar Cells. Inorganics 2018, 6, 52 10.3390/inorganics6020052.

[ref14] Albero J.; Atienzar P.; Corma A.; Garcia H. Efficiency Records in Mesoscopic Dye-Sensitized Solar Cells. Chem. Rec. 2015, 15, 803–828. 10.1002/tcr.201500007. 26183911

[ref15] Vougioukalakis G. C.; Philippopoulos A. I.; Stergiopoulos T.; Falaras P. Contributions to the Development of Ruthenium-Based Sensitizers for Dye-Sensitized Solar Cells. Coord. Chem. Rev. 2011, 2602–2621. 10.1016/j.ccr.2010.11.006.

[ref16] Nazeeruddin M. K.; Kay A.; Rodicio I.; Humphry-Baker R.; Müller E.; Liska P.; Vlachopoulos N.; Grätzel M. Conversion of Light to Electricity by Cis-X2Bis (2,2′-Bipyridyl-4,4′-Dicarboxylate) Ruthenium (II) Charge-Transfer Sensitizers (X = Cl–, Br–, I–, CN–, and SCN−) on Nanocrystalline TiO2 Electrodes. J. Am. Chem. Soc. 1993, 115, 6382–6390. 10.1021/ja00067a063.

[ref17] Nazeeruddin M. K.; Humphry-Baker R.; Liska P.; Grätzel M. Investigation of Sensitizer Adsorption and the Influence of Protons on Current and Voltage of a Dye-Sensitized Nanocrystalline TiO2 Solar Cell. J. Phys. Chem. B 2003, 107, 8981–8987. 10.1021/jp022656f.

[ref18] Nazeeruddin M. K.; Péchy P.; Grätzel M. Efficient Panchromatic Sensitization of Nanocrystalline TiO2 Films by a Black Dye Based on a Trithiocyanato-Ruthenium Complex. Chem. Commun. 1997, 1, 1705–1706. 10.1039/a703277c.

[ref19] Min Park J.; Lee J. H.; Jang W.-D. Applications of Porphyrins in Emerging Energy Conversion Technologies. Coord. Chem. Rev. 2020, 407, 213157 10.1016/j.ccr.2019.213157.

[ref20] Song H.; Liu Q.; Xie Y. Porphyrin-Sensitized Solar Cells: Systematic Molecular Optimization, Coadsorption and Cosensitization. Chem. Commun. 2018, 54, 1811–1824. 10.1039/C7CC09671B. 29372729

[ref21] Yella A.; Lee H. W.; Tsao H. N.; Yi C.; Chandiran A. K.; Nazeeruddin M. K.; Diau E. W. G.; Yeh C. Y.; Zakeeruddin S. M.; Grätzel M. Porphyrin-Sensitized Solar Cells with Cobalt (II/III)-Based Redox Electrolyte Exceed 12 Percent Efficiency. Science 2011, 334, 629–634. 10.1126/science.1209688. 22053043

[ref22] Mathew S.; Yella A.; Gao P.; Humphry-Baker R.; Curchod B. F. E.; Ashari-Astani N.; Tavernelli I.; Rothlisberger U.; Nazeeruddin M. K.; Grätzel M. Dye-Sensitized Solar Cells with 13% Efficiency Achieved through the Molecular Engineering of Porphyrin Sensitizers. Nat. Chem. 2014, 6, 242–247. 10.1038/nchem.1861. 24557140

[ref23] Lu X.; Wei S.; Wu C. M. L.; Li S.; Guo W. Can Polypyridyl Cu(I)-Based Complexes Provide Promising Sensitizers for Dye-Sensitized Solar Cells? A Theoretical Insight into Cu(I) versus Ru(II) Sensitizers. J. Phys. Chem. C 2011, 115, 3753–3761. 10.1021/jp111325y.

[ref24] Magni M.; Biagini P.; Colombo A.; Dragonetti C.; Roberto D.; Valore A. Versatile Copper Complexes as a Convenient Springboard for Both Dyes and Redox Mediators in Dye Sensitized Solar Cells. Coord. Chem. Rev. 2016, 322, 69–93. 10.1016/j.ccr.2016.05.008.

[ref25] Risi G.; Becker M.; Housecroft C. E.; Constable E. C. Are Alkynyl Spacers in Ancillary Ligands in Heteroleptic Bis(Diimine)Copper(I) Dyes Beneficial for Dye Performance in Dye-Sensitized Solar Cells?. Molecules 2020, 25, 1528 10.3390/molecules25071528. PMC7180879 32230862

[ref26] Cao Y.; Saygili Y.; Ummadisingu A.; Teuscher J.; Luo J.; Pellet N.; Giordano F.; Zakeeruddin S. M.; Moser J. E.; Freitag M.; Hagfeldt A.; Grätzel M. 11% Efficiency Solid-State Dye-Sensitized Solar Cells with Copper(II/I) Hole Transport Materials. Nat. Commun. 2017, 8, 15390 10.1038/ncomms15390. 28598436 PMC5472710

[ref27] Saygili Y.; Söderberg M.; Pellet N.; Giordano F.; Cao Y.; Munoz-García A. B.; Zakeeruddin S. M.; Vlachopoulos N.; Pavone M.; Boschloo G.; Kavan L.; Moser J. E.; Grätzel M.; Hagfeldt A.; Freitag M. Copper Bipyridyl Redox Mediators for Dye-Sensitized Solar Cells with High Photovoltage. J. Am. Chem. Soc. 2016, 138, 15087–15096. 10.1021/jacs.6b10721. 27749064

[ref28] Colombo A.; Di Carlo G.; Dragonetti C.; Magni M.; Orbelli Biroli A.; Pizzotti M.; Roberto D.; Tessore F.; Benazzi E.; Bignozzi C. A.; Casarin L.; Caramori S. Coupling of Zinc Porphyrin Dyes and Copper Electrolytes: A Springboard for Novel Sustainable Dye-Sensitized Solar Cells. Inorg. Chem. 2017, 56, 14189–14197. 10.1021/acs.inorgchem.7b02323. 29091412

[ref29] Higashino T.; Iiyama H.; Nishimura I.; Imahori H. Exploration on the Combination of Push-Pull Porphyrin Dyes and Copper(I/II) Redox Shuttles toward High-Performance Dye-Sensitized Solar Cells. Chem. Lett. 2020, 49, 936–939. 10.1246/cl.200317.

[ref30] Alonso-Vante N.; Nierengarten J.-F.; Sauvage J.-P. Spectral Sensitization of Large-Band-Gap Semiconductors (Thin Films and Ceramics) by a Carboxylated Bis(1,10-Phenanthroline)Copper(I) Complex. J. Chem. Soc., Dalton Trans. 1994, 97, 1649 10.1039/dt9940001649.

[ref31] Huang J.; Buyukcakir O.; Mara M. W.; Coskun A.; Dimitrijevic N. M.; Barin G.; Kokhan O.; Stickrath A. B.; Ruppert R.; Tiede D. M.; Stoddart J. F.; Sauvage J. P.; Chen L. X. Highly Efficient Ultrafast Electron Injection from the Singlet MLCT Excited State of Copper(I) Diimine Complexes to TiO2 Nanoparticles. Angew. Chem., Int. Ed. 2012, 51, 12711–12715. 10.1002/anie.201204341. 23136096

[ref32] Sandroni M.; Pellegrin Y.; Odobel F. Heteroleptic Bis-Diimine Copper(I) Complexes for Applications in Solar Energy Conversion. C. R. Chim. 2016, 19, 79–93. 10.1016/j.crci.2015.06.008.

[ref33] Bessho T.; Constable E. C.; Graetzel M.; Hernandez Redondo A.; Housecroft C. E.; Kylberg W.; Nazeeruddin M. K.; Neuburger M.; Schaffner S. An Element of Surprise: Efficient Copper-Functionalized Dye-Sensitized Solar Cells. Chem. Commun. 2008, 3717 10.1039/b808491b. 18685754

[ref34] Bozic-Weber B.; Brauchli S. Y.; Constable E. C.; Fürer S. O.; Housecroft C. E.; Malzner F. J.; Wright I. A.; Zampese J. A. Improving the Photoresponse of Copper(i) Dyes in Dye-Sensitized Solar Cells by Tuning Ancillary and Anchoring Ligand Modules. Dalton Trans. 2013, 42, 12293–12308. 10.1039/c3dt51416a. 23851470

[ref35] Malzner F. J.; Brauchli S. Y.; Constable E. C.; Housecroft C. E.; Neuburger M. Halos Show the Path to Perfection: Peripheral Iodo-Substituents Improve the Efficiencies of Bis(Diimine)Copper(i) Dyes in DSCs. RSC Adv. 2014, 4, 48712–48723. 10.1039/C4RA06823H.

[ref36] Brauchli S. Y.; Malzner F. J.; Constable E. C.; Housecroft C. E. Copper(i)-Based Dye-Sensitized Solar Cells with Sterically Demanding Anchoring Ligands: Bigger Is Not Always Better. RSC Adv. 2015, 5, 48516–48525. 10.1039/C5RA07449E.

[ref37] Dragonetti C.; Magni M.; Colombo A.; Melchiorre F.; Biagini P.; Roberto D. Coupling of a Copper Dye with a Copper Electrolyte: A Fascinating Springboard for Sustainable Dye-Sensitized Solar Cells. ACS Appl. Mater. Interfaces 2018, 1, 751–756. 10.1021/acsaem.7b00196.

[ref38] Karpacheva M.; Malzner F. J.; Wobill C.; Büttner A.; Constable E. C.; Housecroft C. E. Cuprophilia: Dye-Sensitized Solar Cells with Copper(I) Dyes and Copper(I)/(II) Redox Shuttles. Dyes Pigm. 2018, 156, 410–416. 10.1016/j.dyepig.2018.04.033.

[ref39] Leandri V.; Pizzichetti A. R. P.; Xu B.; Franchi D.; Zhang W.; Benesperi I.; Freitag M.; Sun L.; Kloo L.; Gardner J. M. Exploring the Optical and Electrochemical Properties of Homoleptic versus Heteroleptic Diimine Copper(I) Complexes. Inorg. Chem. 2019, 58, 12167–12177. 10.1021/acs.inorgchem.9b01487. 31483631

[ref40] Kaeser A.; Delavaux-Nicot B.; Duhayon C.; Coppel Y.; Nierengarten J.-F. Heteroleptic Silver(I) Complexes Prepared from Phenanthroline and Bis-Phosphine Ligands. Inorg. Chem. 2013, 52, 14343–14354. 10.1021/ic402342y. 24279392

[ref41] Scaltrito D. V.; Thompson D. W.; O’Callaghan J. A.; Meyer G. J. MLCT Excited States of Cuprous Bis-Phenanthroline Coordination Compounds. Coord. Chem. Rev. 2000, 208, 243–266. 10.1016/S0010-8545(00)00309-X.

[ref42] Leandri V.; Daniel Q.; Chen H.; Sun L.; Gardner J. M.; Kloo L. Electronic and Structural Effects of Inner Sphere Coordination of Chloride to a Homoleptic Copper(II) Diimine Complex. Inorg. Chem. 2018, 57, 4556–4562. 10.1021/acs.inorgchem.8b00225. 29608296

[ref43] Schmittel M.; Ganz A. Stable Mixed Phenanthroline Copper(i) Complexes. Key Building Blocks for Supramolecular Coordination Chemistry. Chem. Commun. 1997, 999–1000. 10.1039/a701509g.

[ref44] Miller M. T.; Gantzel P. K.; Karpishin T. B. A Highly Emissive Heteroleptic Copper(I) Bis(Phenanthroline) Complex: [Cu(Dbp)(Dmp)]+ (Dbp = 2,9-Di- Tert -Butyl-1,10-Phenanthroline; Dmp = 2,9-Dimethyl-1,10-Phenanthroline). J. Am. Chem. Soc. 1999, 121, 4292–4293. 10.1021/ja9901415.

[ref45] Sandroni M.; Kayanuma M.; Planchat A.; Szuwarski N.; Blart E.; Pellegrin Y.; Daniel C.; Boujtita M.; Odobel F. First Application of the HETPHEN Concept to New Heteroleptic Bis(Diimine) Copper(i) Complexes as Sensitizers in Dye Sensitized Solar Cells. Dalton Trans. 2013, 42, 10818–10827. 10.1039/c3dt50852h. 23783812

[ref46] Sandroni M.; Favereau L.; Planchat A.; Akdas-Kilig H.; Szuwarski N.; Pellegrin Y.; Blart E.; Le Bozec H.; Boujtita M.; Odobel F. Heteroleptic Copper(i)-Polypyridine Complexes as Efficient Sensitizers for Dye Sensitized Solar Cells. J. Mater. Chem. A 2014, 2, 9944–9947. 10.1039/c4ta01755b.

[ref47] Kabehie S.; Stieg A. Z.; Xue M.; Liong M.; Wang K. L.; Zink J. I. Surface Immobilized Heteroleptic Copper Compounds as State Variables That Show Negative Differential Resistance. J. Phys. Chem. Lett. 2010, 1, 589–593. 10.1021/jz900324f.

[ref48] Housecroft C. E.; Constable E. C. The Emergence of Copper(I)-Based Dye Sensitized Solar Cells. Chem. Soc. Rev. 2015, 44, 8386–8398. 10.1039/c5cs00215j. 26356386

[ref49] Frisch M. J.; Trucks G. W.; Schlegel H. B.; Scuseria G. E.; Robb M. A.; Cheeseman J. R.; Scalmani G.; Barone V.; Petersson G. A.; Nakatsuji H.; Li X.; Caricato M.; Marenich A. V.; Bloino J.; Janesko B. G.; Gomperts R.; Mennucci B.; Hratchian H. P.; Ortiz J. V.; Izmaylov A. F.; Sonnenberg J. L.; Williams-Young D.; Ding F.; Lipparini F.; Egidi F.; Goings J.; Peng B.; Petrone A.; Henderson T.; Ranasinghe D.; Zakrzewski V. G.; Gao J.; Rega N.; Zheng G.; Liang W.; Hada M.; Ehara M.; Toyota K.; Fukuda R.; Hasegawa J.; Ishida M.; Nakajima T.; Honda Y.; Kitao O.; Nakai H.; Vreven T.; Throssell K.; Montgomery J. A. Jr.; Peralta J. E.; Ogliaro F.; Bearpark M. J.; Heyd J. J.; Brothers E. N.; Kudin K. N.; Staroverov V. N.; Keith T. A.; Kobayashi R.; Normand J.; Raghavachari K.; Rendell A. P.; Burant J. C.; Iyengar S. S.; Tomasi J.; Cossi M.; Millam J. M.; Klene M.; Adamo C.; Cammi R.; Ochterski J. W.; Martin R. L.; Morokuma K.; Farkas O.; Foresman J. B.; Fox D. J. Gaussian 16, revision B.01; Gaussian, Inc.: Wallingford CT, 2016.

[ref50] Yanai T.; Tew D. P.; Handy N. C. A New Hybrid Exchange-Correlation Functional Using the Coulomb-Attenuating Method (CAM-B3LYP). Chem. Phys. Lett 2004, 393, 51–57. 10.1016/j.cplett.2004.06.011.

[ref51] Figgen D.; Rauhut G.; Dolg M.; Stoll H. Energy-Consistent Pseudopotentials for Group 11 and 12 Atoms: Adjustment to Multi-Configuration Dirac–Hartree–Fock Data. Chem. Phys. 2005, 311, 227–244. 10.1016/j.chemphys.2004.10.005.

[ref52] Peterson K. A. Systematically Convergent Basis Sets with Relativistic Pseudopotentials. I. Correlation Consistent Basis Sets for the Post- d Group 13–15 Elements. J. Chem. Phys. 2003, 119, 11099–11112. 10.1063/1.1622923.

[ref53] Schäfers F. The Crystal Monochromator Beamline KMC-1 at BESSY II. J. Large-Scale Res. Facil. JLSRF 2016, 2, A96 10.17815/jlsrf-2-92.

[ref54] Johansson E. M. J.; Hedlund M.; Siegbahn H.; Rensmo H. Electronic and Molecular Surface Structure of Ru(Tcterpy)(NCS)3 and Ru(Dcbpy)2(NCS)2 Adsorbed from Solution onto Nanostructured TiO2: A Photoelectron Spectroscopy Study. J. Phys. Chem. B 2005, 109, 22256–22263. 10.1021/jp0525282. 16853898

[ref55] Ida T.; Ando M.; Toraya H. Extended Pseudo-Voigt Function for Approximating the Voigt Profile. J. Appl. Crystallogr. 2000, 33, 1311–1316. 10.1107/S0021889800010219.

[ref56] Scofield J. H. Theoretical photoionization cross sections from 1 to 1500 keV; California University, 1973; p 5–6.

[ref57] Boschloo G.; Hagfeldt A. Characteristics of the Iodide/Triiodide Redox Mediator in Dye-Sensitized Solar Cells. Acc. Chem. Res. 2009, 42, 1819–1826. 10.1021/ar900138m. 19845388

[ref58] Franke R.; Chassé T.; Streubel P.; Meisel A. Auger Parameters and Relaxation Energies of Phosphorus in Solid Compounds. J. Electron Spectrosc. Relat. Phenom. 1991, 56, 381–388. 10.1016/0368-2048(91)85035-R.

[ref59] Moulder J. F.; Stickle W. F.; Sobol P. E.; Bomben K. D. Handbook of X-ray Photoelectron Spectroscopy: A Reference Book of Standard Spectra for Identification and Interpretation of XPS Data; Physical Electronics, Inc., 1992.

[ref60] Colombo A.; Dragonetti C.; Roberto D.; Valore A.; Biagini P.; Melchiorre F. A Simple Copper(I) Complex and Its Application in Efficient Dye Sensitized Solar Cells. Inorg. Chim. Acta 2013, 407, 204–209. 10.1016/j.ica.2013.07.028.

[ref61] Kaeser A.; Delavaux-Nicot B.; Duhayon C.; Coppel Y.; Nierengarten J. F. Heteroleptic Silver(I) Complexes Prepared from Phenanthroline and Bis-Phosphine Ligands. Inorg. Chem. 2013, 52, 14343–14354. 10.1021/ic402342y. 24279392

[ref62] De Angelis F.; Fantacci S.; Mosconi E.; Nazeeruddin M. K.; Grätzel M. Absorption Spectra and Excited State Energy Levels of the N719 Dye on TiO2 in Dye-Sensitized Solar Cell Models. J. Phys. Chem. C 2011, 115, 8825–8831. 10.1021/jp111949a.

[ref63] Murali M. G.; Wang X.; Wang Q.; Valiyaveettil S. Design and Synthesis of New Ruthenium Complex for Dye-Sensitized Solar Cells. RSC Adv. 2016, 6, 57872–57879. 10.1039/C6RA10881D.

[ref64] Ji J.-M.; Zhou H.; Kim H. K. Rational Design Criteria for D−π–A Structured Organic and Porphyrin Sensitizers for Highly Efficient Dye-Sensitized Solar Cells. J. Mater. Chem. A 2018, 6, 14518–14545. 10.1039/C8TA02281J.

[ref65] Yang J.; Ganesan P.; Teuscher J.; Moehl T.; Kim Y. J.; Yi C.; Comte P.; Pei K.; Holcombe T. W.; Nazeeruddin M. K.; Hua J.; Zakeeruddin S. M.; Tian H.; Grätzel M. Influence of the Donor Size in D-π-A Organic Dyes for Dye-Sensitized Solar Cells. J. Am. Chem. Soc. 2014, 136, 5722–5730. 10.1021/ja500280r. 24655036

[ref66] Lu J.; Liu S.; Wang M. Push-Pull Zinc Porphyrins as Light-Harvesters for Efficient Dye-Sensitized Solar Cells. Front. Chem. 2018, 6, 541 10.3389/fchem.2018.00541. 30519554 PMC6251255

[ref67] Risi G.; Becker M.; Housecroft C. E.; Constable E. C. Are Alkynyl Spacers in Ancillary Ligands in Heteroleptic Bis(Diimine)Copper(I) Dyes Beneficial for Dye Performance in Dye-Sensitized Solar Cells?. Molecules 2020, 25, 1528 10.3390/molecules25071528. PMC7180879 32230862

[ref68] McCusker C. E.; Castellano F. N. Design of a Long-Lifetime, Earth-Abundant, Aqueous Compatible Cu(I) Photosensitizer Using Cooperative Steric Effects. Inorg. Chem. 2013, 52, 8114–8120. 10.1021/ic401213p. 23789625

[ref69] Ruthkosky M.; Castellano F. N.; Meyer G. J. Photodriven Electron and Energy Transfer from Copper Phenanthroline Excited States. Inorg. Chem. 1996, 35, 6406–6412. 10.1021/ic960503z. 11666787

