# Supplementary material for: Effect of the Ancillary Ligand on the Performance of Heteroleptic Cu(I) Diimine Complexes as Dyes in Dye-Sensitized Solar Cells
Source: ACS Appl Energy Mater. 2022 Jan 13;5(2):1460–70. doi: 10.1021/acsaem.1c02778 (PMC8889538; doi:10.1021/acsaem.1c02778)
Supplement: Supplementary file 1 — ae1c02778_si_001.pdf [file ae1c02778_si_001.pdf]

Supporting Information

Effect of the Ancillary Ligand on the Performance of Heteroleptic Cu(I) Diimine Complexes Dyes in Dye-sensitized Solar Cells

Daniele Franchi,[a,b] Valentina Leandri,[c] Angela Raffaella Pia Pizzichetti,[c] Bo Xu,[d] Yan Hao,[c] Wei Zhang,[c] Tamara Sloboda,[c] Sebastian Svanström,[e] Ute B. Cappel,[c] Lars Kloo,[c] Licheng Sun,[b,f] James M. Gardner*[c]

[a] Institute of Chemistry of Organometallic Compounds (CNR-ICCOM), Via Madonna del Piano 10, 50019 Sesto Fiorentino, Italy.

[b] Division of Organic Chemistry, Centre of Molecular Devices, Department of Chemistry, KTH Royal Institute of Technology, SE-10044, Stockholm, Sweden.

[c] Division of Applied Physical Chemistry, Centre of Molecular Devices, Department of Chemistry, KTH Royal Institute of Technology, SE-10044, Stockholm, Sweden. E-mail: jgardner@kth.se

[d] Division of Physical Chemistry, Centre of Molecular Devices, Department of Chemistry, Ångström Laboratory, Uppsala University, Box 523, SE-75120 Uppsala, Sweden.

[e] Division of X-ray Photon Science, Department of Physics and Astronomy, Uppsala University, Box 516, SE-751 20, Uppsala, Sweden

[f] Center of Artificial Photosynthesis for Solar Fuels, School of Science, Westlake University, Hangzhou 310024, China.

Additional HAXPES data and pseudo-Voigt function fitting

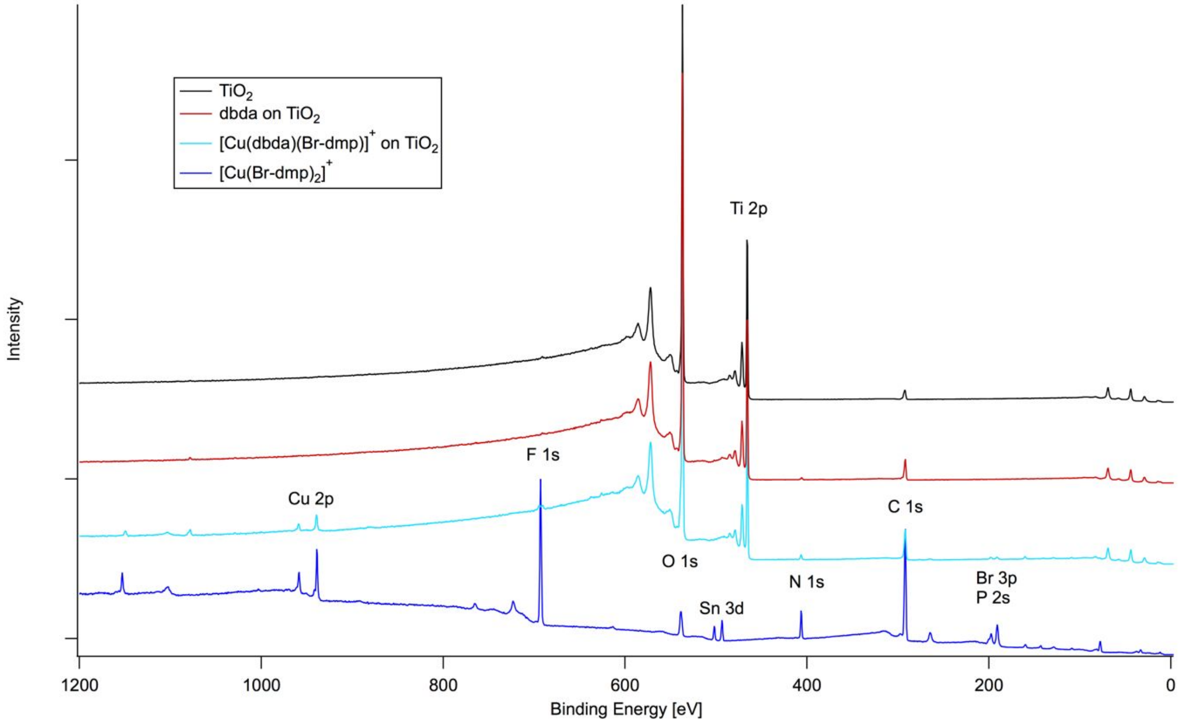

Figure S1 HAXPES overview spectra measured with a photon energy of 3000 eV and a pass energy of 500 eV.

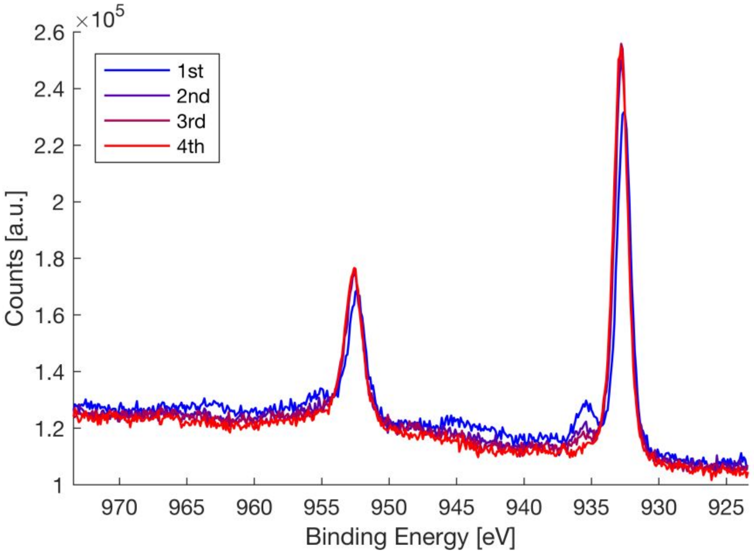

Figure S2 Cu 2p spectra measured for $[Cu(Br\text{-}dmp)_2]^+$ on FTO with a time interval of approximately 20 minutes of X-ray illumination between spectra. The first spectrum was measured within 10 minutes of X-ray illumination of the sample spot. Spectra are binding energy calibrated against an external gold reference. The main peak at 932 eV can be assigned to Cu $2p_{3/2}$ of Cu(I) and the peak at 952 eV to Cu $2p_{1/2}$ of Cu(I). The smaller peak at 936 eV can be assigned to Cu $2p_{3/2}$ of Cu(II) and the extra intensity around 940 eV in the first spectrum to satellite features associated with Cu(II). Under X-ray illumination conversion of Cu(II) to Cu(I) is observed. The initial shift of the Cu(I) position to higher binding energies is assigned to sample charging during X-ray illumination. Due to the charging, the 2nd spectrum is used in all figures in the main manuscript.

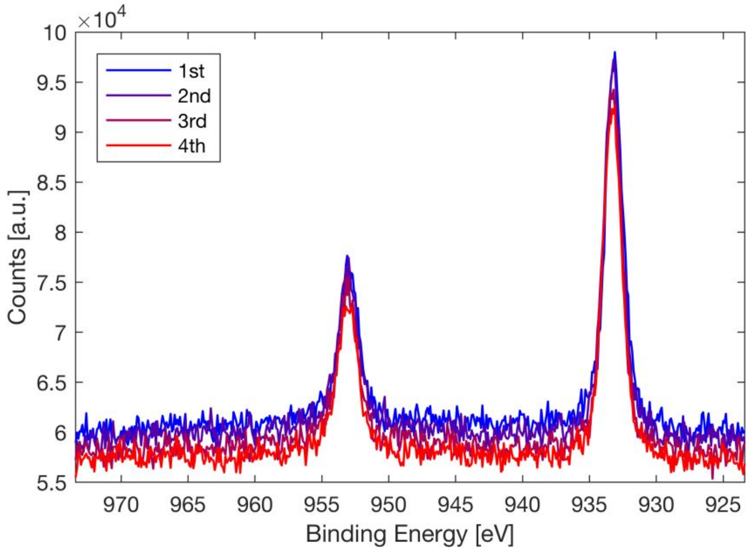

Figure S3 Cu 2p spectra measured for $[Cu(dbda)(Br\text{-}dmp)]^+$ on TiO_2 with a time interval of approximately 20 minutes between spectra. The first spectrum was measured within 10 minutes of X-ray illumination of the sample spot. The change in background intensity is assigned to variations in X-ray intensity and initial, small shift to higher binding energies is assigned to sample charging during X-ray illumination. An average of spectra 2 to 4 is shown in the main manuscript.

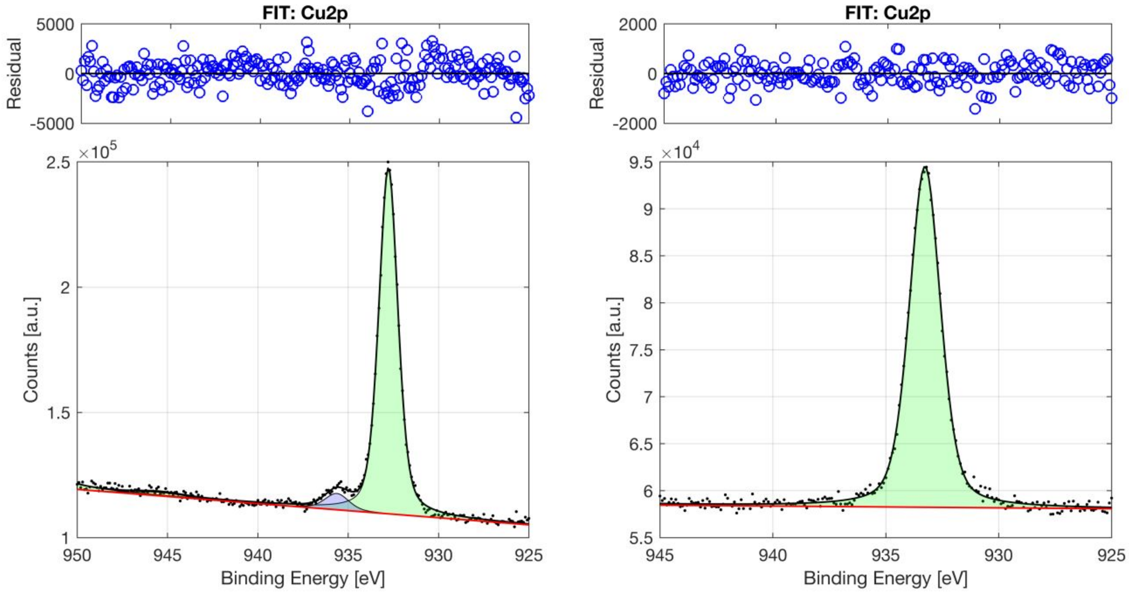

Figure S4 Cu $3p_{3/2}$ spectra of $[Cu(Br\text{-}dmp)_2]^+$ on FTO (left) and $[Cu(dbda)(Br\text{-}dmp)]^+$ on TiO_2 (right) with curve fit to pseudo-Voigt functions. Spectra are binding energy calibrated against an external gold

reference. For $[Cu(Br\text{-}dmp)_2]^+$, the Cu 2p measured after approximately 25 minutes of X-ray illumination was fitted. For $[Cu(dbda)(Br\text{-}dmp)]^+$, and average of spectra from iterations 2 to 4 is shown and fitted.

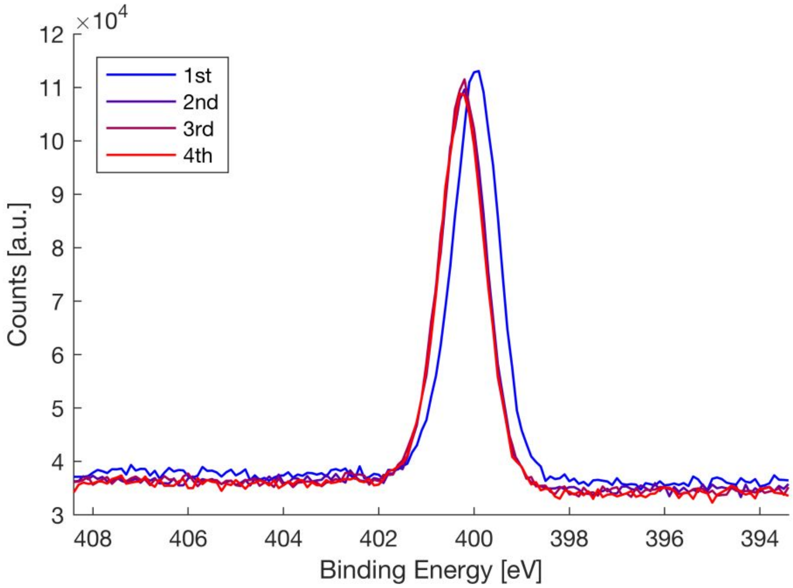

Figure S5 N 1s spectra measured for $[Cu(Br\text{-}dmp)_2]^+$ on FTO with a time interval of approximately 20 minutes between spectra. The first spectrum was measured within 5 minutes of X-ray illumination of the sample spot. Spectra are binding energy calibrated against an external gold reference. The initial shift of the N 1s position to higher binding energies is assigned to sample charging during X-ray illumination. Due to the charging, the 2nd spectrum is used in all figures in the main manuscript and in the fitting shown below.

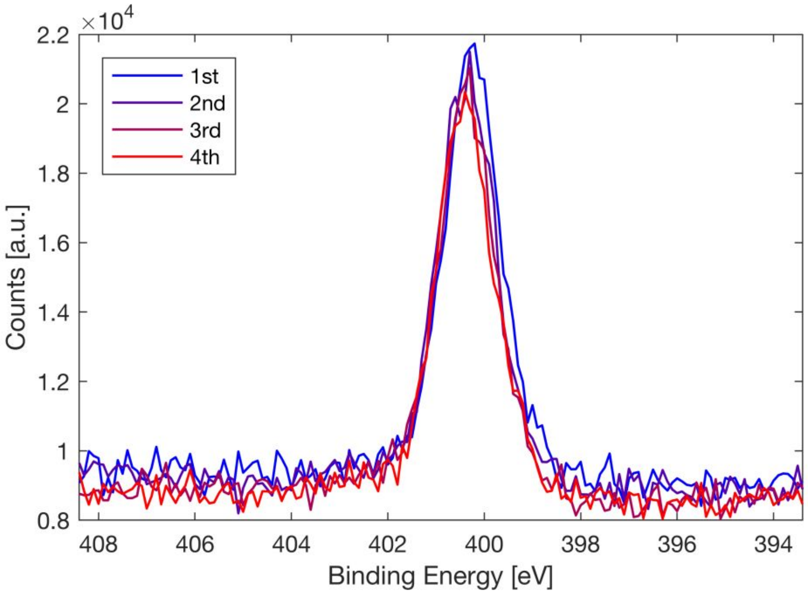

Figure S6 N 1s spectra measured for [Cu(dbda)(Br-dmp)]$^+$ on TiO_2 with a time interval of approximately 20 minutes between spectra. The first spectrum was measured within 5 minutes of X-ray illumination of the sample spot. The change in background intensity is assigned to variations in X-ray intensity and the initial, small shift to higher binding energies is assigned to sample charging during X-ray illumination. An average of spectra 2 to 4 is shown in the main manuscript and in the fitting shown below.

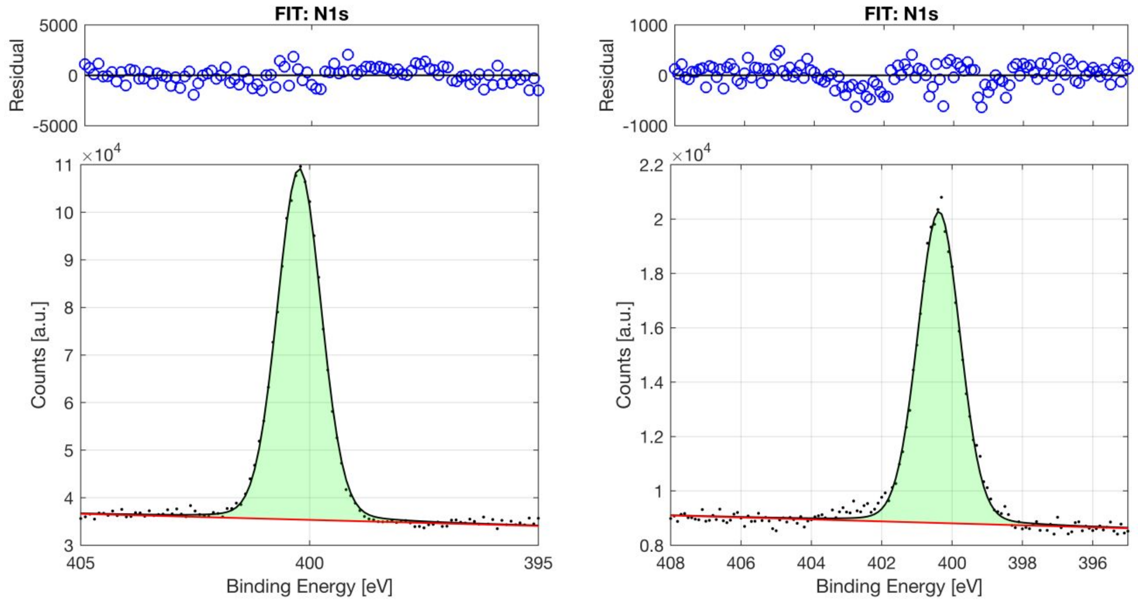

Figure S7 N 1s spectra of [Cu(Br-dmp)$_2$]$^+$ on FTO (left) and [Cu(dbda)(Br-dmp)]$^+$ on TiO_2 (right) with curve fit to a pseudo-Voigt function. Spectra are binding energy calibrated against an external gold reference.

For $[Cu(Br\text{-}dmp)_2]^+$, the N 1s measured after approximately 20 minute X-ray illumination was fitted. For $[Cu(dbda)(Br\text{-}dmp)]^+$, and average of spectra from iterations 2 to 4 is shown and fitted.

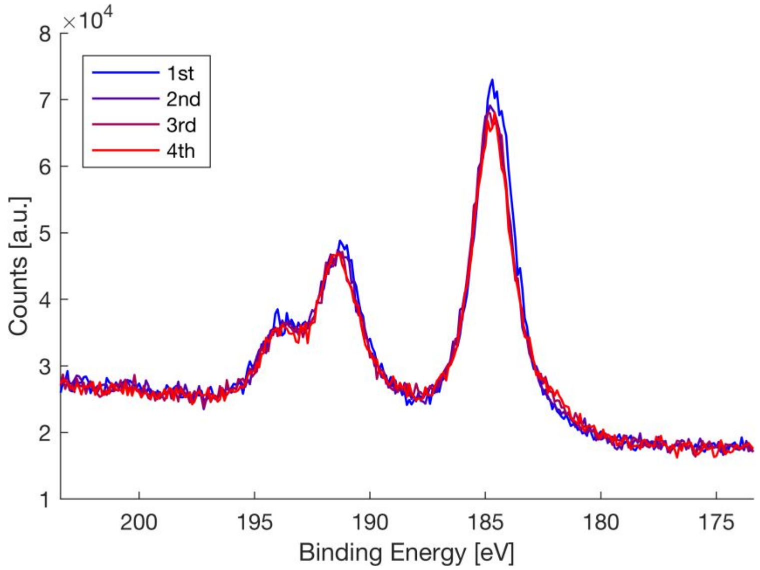

Figure S8 Br 3p and P 2s spectra measured for $[Cu(Br\text{-}dmp)_2]^+$ on FTO with a time interval of approximately 20 minutes between spectra. The first spectrum was measured within 15 minutes of X-ray illumination of the sample spot. Spectra are binding energy calibrated against an external gold reference. A small increase in signal is observed at 182 eV over time, which can be assigned to the instability of Br-dmp under X-ray illumination and the formation of bromide ions. Due to sample charging, the 2nd spectrum is used in all figures in the main manuscript and in the fitting shown below.

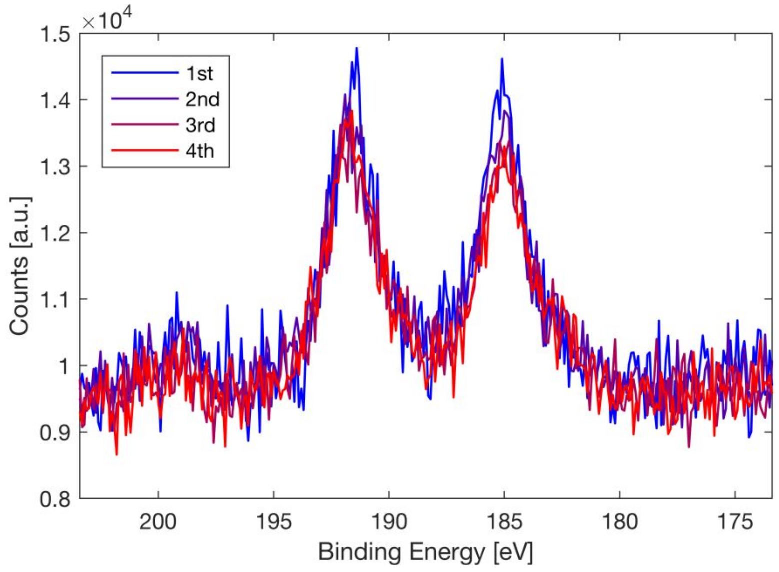

Figure S9 Br 3p and P 2s spectra measured for [Cu(dbda)(Br-dmp)]$^+$ on TiO2 with a time interval of approximately 20 minutes between spectra. The first spectrum was measured within 15 minutes of X-ray illumination of the sample spot. An average of spectra 2 to 4 is shown in the main manuscript and in the fitting shown below.

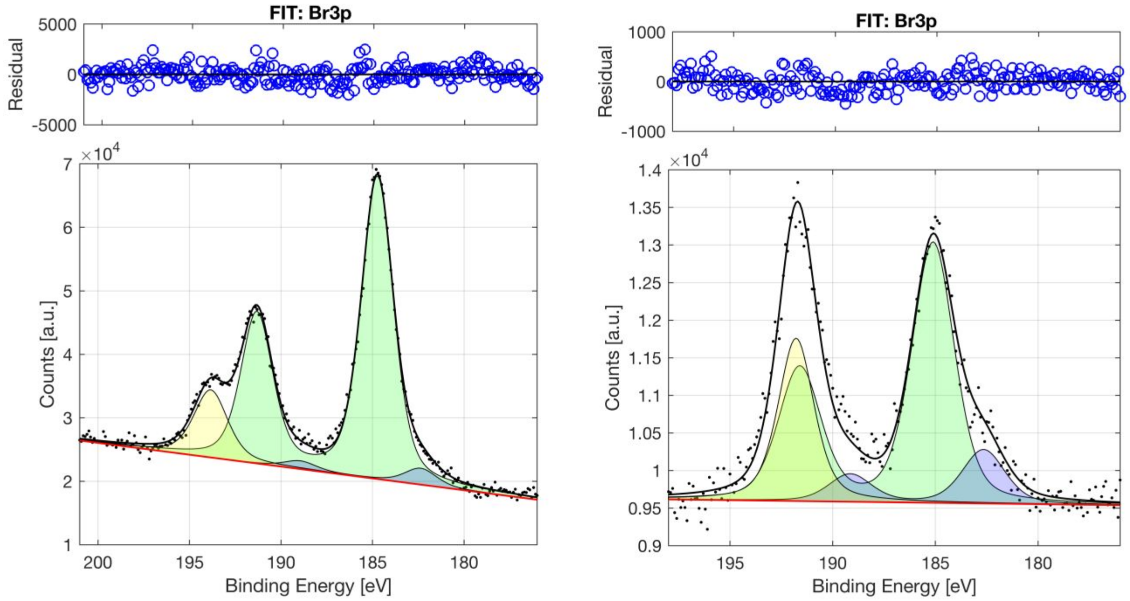

Figure S10 Br 3p and P 2s spectra of $[Cu(Br\text{-}dmp)_2]^+$ on FTO (left) and [Cu(dbda)(Br-dmp)]$^+$ on TiO_2 (right) with curve fit to a series of pseudo-Voigt function. The doublet shown in green represents the main Br 3p peaks from Br-dmp. The smaller doublet at lower binding energies is assigned to Br^-, which forms under

X-ray illumination. As the total Br intensity of the sample does not change over time with X-ray illumination, this peak is included in the quantification of the Br 3p spectra against N 1s. The singlet in yellow is assigned to P 2s and appears at different binding energies for $[Cu(Br\text{-}dmp)_2]^+$ and $[Cu(dbda)(Br\text{-}dmp)]^+$ in agreement with the observations for P 1s shown in the main manuscript. For the heteroleptic complex, the phosphorous peaks are chemically shifted to lower binding energies and therefore the P 2s peak overlaps directly with the Br $3p_{1/2}$ peak explaining the different spectral shapes observed for the homo- and heteroleptic complexes. The spectra are binding energy calibrated against an external gold reference. For $[Cu(Br\text{-}dmp)_2]^+$, the spectrum was measured after approximately 30 minute X-ray illumination was fitted. For $[Cu(dbda)(Br\text{-}dmp)]^+$, and average of spectra from iterations 2 to 4 is shown and fitted.

Table S1 Quantification based on photoemission cross-sections and core level fitting for $[Cu(Br\text{-}dmp)_2]^+$ relative to N 1s intensity for the different repeat measurements of the N 1s core level.

$[Cu(Br\text{-}dmp)_2]^+$	N 1s	Br 3p (Br-dmp)	Br 3p (Br^-)	Br 3p (total)	Cu 2p (total)
1st	1.0	0.47	0.01	0.48	0.22
2nd	1.0	0.47	0.03	0.50	0.24
3rd	1.0	0.46	0.04	0.50	0.25
4th	1.0	0.46	0.04	0.50	0.25

Table S2 Quantification based on photoemission cross-sections and core level fitting for $[Cu(dbda)(Br\text{-}dmp)]^+$ relative to N 1s intensity for the different repeat measurements of the N 1s core level.

$[Cu(dbda)(Br\text{-}dmp)]^+$	N 1s	Br 3p (Br-dmp)	Br 3p (Br^-)	Br 3p (total)	Cu 2p (total)
1st	1.0	0.23	0.03	0.26	0.38
2nd	1.0	0.21	0.04	0.25	0.39
3rd	1.0	0.21	0.05	0.26	0.39
4th	1.0	0.20	0.05	0.25	0.40

Ground state geometry and spatial disposition of frontier orbitals obtained from DFT computation

$[Cu(dbda)_2]^+$		
**Ground state geometry**		
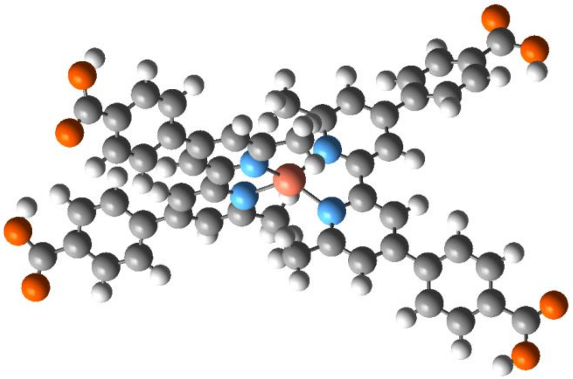		
$[Cu(dbda)(dmp)]^+$		
**Ground state geometry**	**HOMO**	**LUMO**
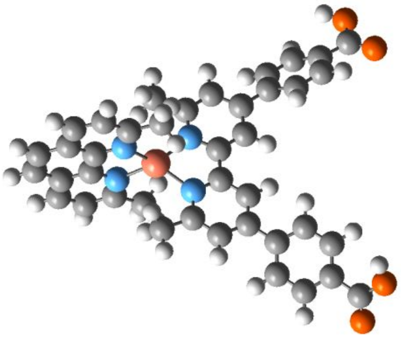	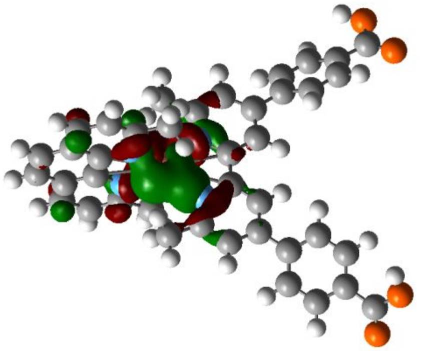	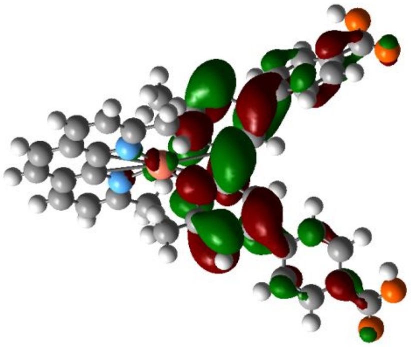
$[Cu(dbda)(Br\text{-}dmp)]^+$		
**Ground state geometry**	**HOMO**	**LUMO**
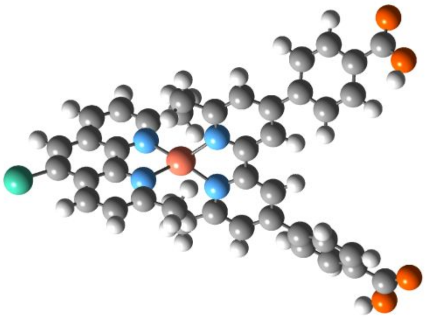	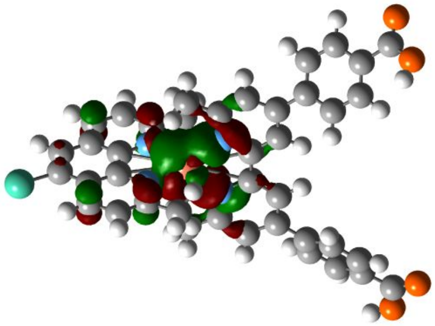	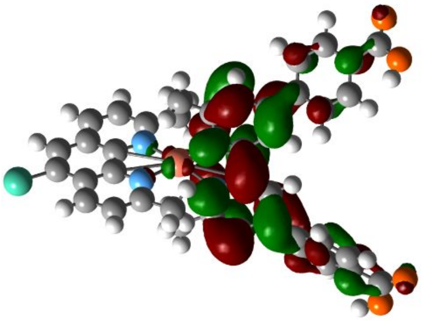
$[Cu(dbda)(dsbtmp)]^+$		
**Ground state geometry**	**HOMO**	**LUMO**

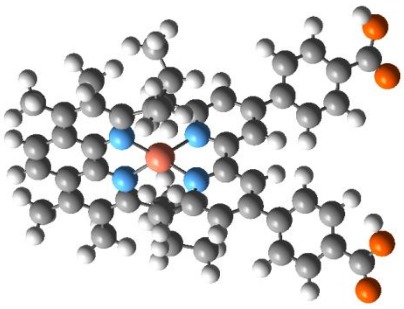	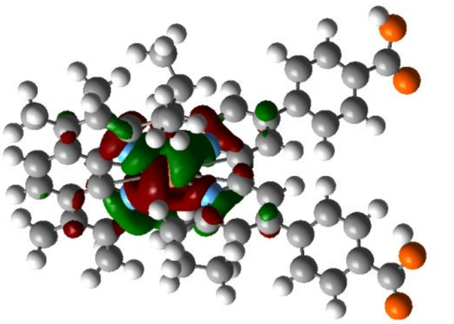	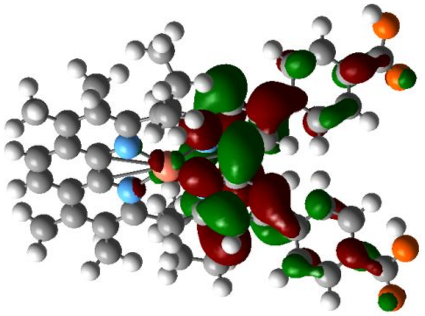
	$[Cu(dbda)(bcp)]^+$	
	**Ground state geometry**	
	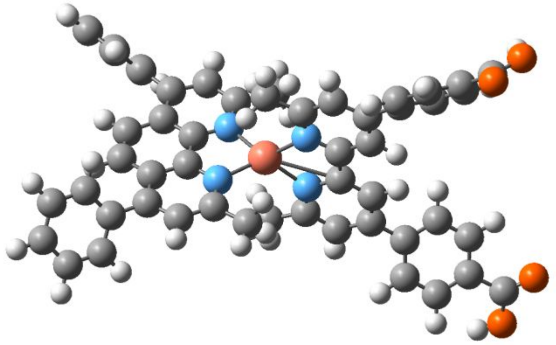	
	$[Cu(dbda)(biq)]^+$	
	**Ground state geometry**	
	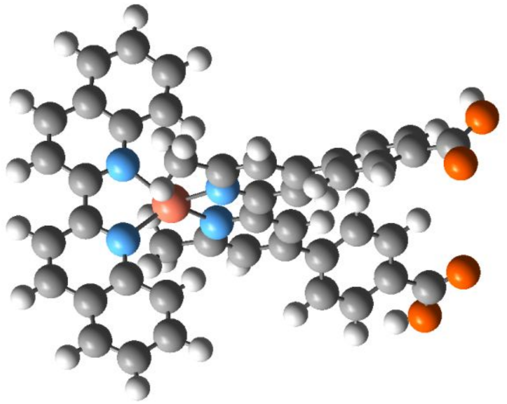	
	$[Cu(dbda)(dap)]^+$	
**Ground state geometry**	**HOMO**	**LUMO**

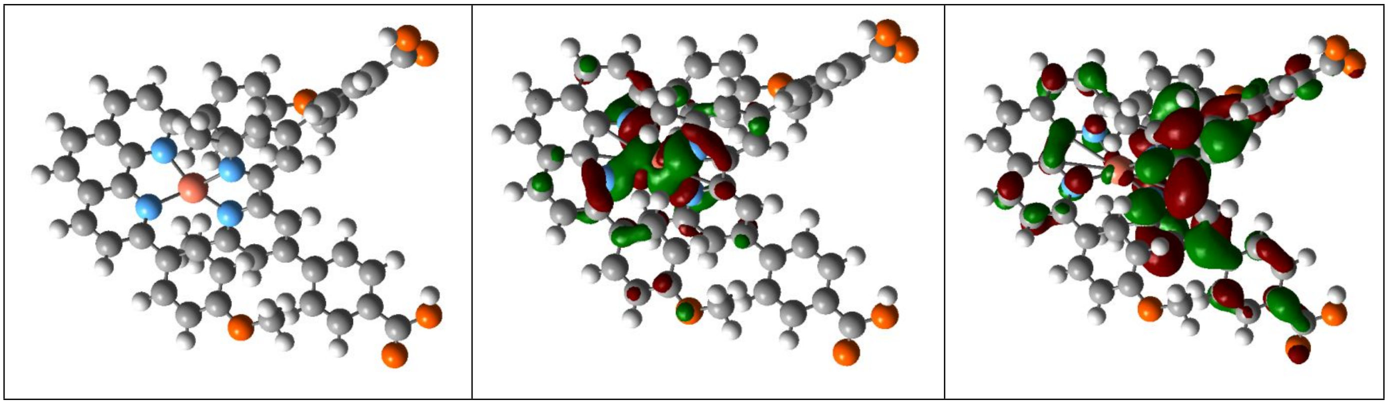

Figure S11 Ground state geometries, HOMO and LUMOs of the Cu(I) dyes in this study calculated by DFT.

Photovoltaic characterization of the DSSC with Co-based electrolytes

Table S3 Detailed photovoltaic parameters of non-optimized electrodes DSSCs (old, diluted paste, total thickness around 7-8 μm) based on I^-/I_3^- and $[Co(bpy)_3]^{2+/3+}$ redox mediators. The dye used is the complex $[Cu(dbda)(bcp)]^+$.

Redox couple	η (%)	V_{OC} (mV)	J_{SC} (mA cm-2)	FF (%)
I^-/I_3^-	0.117 ± 0.03	512 ± 15	0.351 ± 0.05	65 ±3
A $[Co(bpy)_3]^{2+/3+}$	0.052 ± 0.02	485 ± 10	0.199 ± 0.04	53 ± 8
B $[Co(bpy)_3]^{2+/3+}$	0.005 ± 0.001	343 ± 30	0.030 ± 0.006	47 ± 1

The electrolyte compositions are the following. I^-/I_3^-: 0.1 M LiI, 0.05 M I_2, 0.5 M 1-Methylbenzimidazole, 0.6 M 1-Butyl-3-methylimidazolium iodide in 3-methoxypropionitrile. $[Co(bpy)_3]^{2+/3+}$ composition A: Co^{2+} 0.22 M, Co^{3+} 0.05 M, TBP 0.20 M, $LiClO_4$ 0.1 M in acetonitrile; $[Co(bpy)_3]^{2+/3+}$ composition B: Co^{2+} 0.22 M, Co^{3+} 0.05 M, $LiClO_4$ 0.1 M in acetonitrile.

The DSSCs fabricated with the iodide-triiodide electrolyte showed moderate bleaching, most likely due to the solvent, 3-methoxypropionitrile. The DSSCs fabricated with the cobalt electrolyte were significantly bleached directly after electrolyte injection. The electrolyte composition B, which does not include TBP, led to slightly less bleached working electrodes. However, the overall performance of the solar cells was anyway lower.

Table S4 Detailed photovoltaic parameters of optimized electrodes DSSCs based on I^-/I_3^- and dye $[Cu(dbda)(bcp)]^+$.

Electrolyte	η (%)	V_{OC} (mV)	J_{SC} (mA cm-2)	FF (%)
A	1.02 ± 0.05	560 ± 5	2.664 ± 0.03	69 ± 3
B	2.10 ± 0.02	605 ± 5	4.711 ± 0.05	74 ± 0

The electrolyte compositions are the following. A: 0.1 M LiI, 0.05 M I_2, 0.5 M 1-Methylbenzimidazole, 0.6 M 1-Butyl-3-methylimidazolium iodide in 3-methoxypropionitrile; B: 0.025 M LiI, 0.04 M I_2, 0.28 M TBP, 0.65 M 1-Butyl-3-methylimidazolium iodide in 15/85 (v/v) mixture of valeronitrile/acetonitrile.

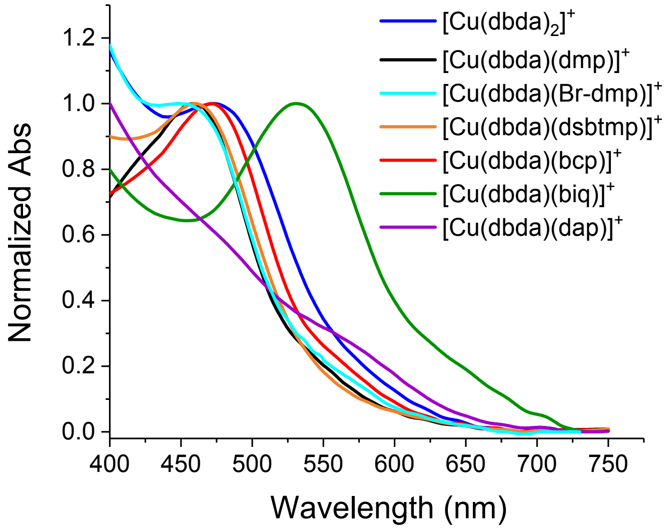

Figure S12 Normalized electronic absorption spectra of the Cu(I) complexes in this work adsorbed on the surface of a thin film of mesoporous anatase TiO_2.

Visual comparison between the DSSCs fabricated in this study

a)

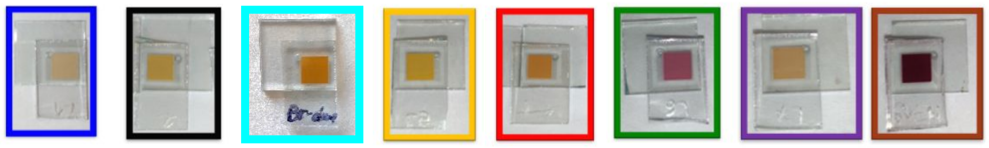

b)

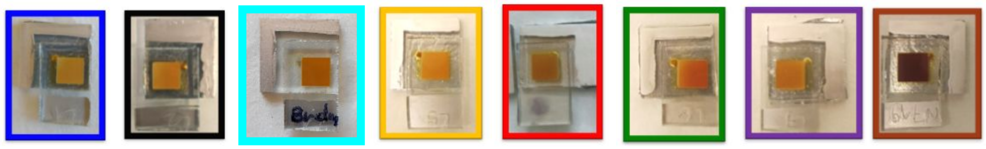

Figure S13. DSSCs based on the Cu(I) dyes in this study and the benchmark dye N719 before (a) and after (b) electrolyte injection. The frame colours correspond, from left to right, to: $[Cu(dbda)_2]^+$ (blue), $[Cu(dbda)(dmp)]^+$ (black), $[Cu(dbda)(Br\text{-}dmp)_2]^+$ (cyan), $[Cu(dbda)(dsbtmp)]^+$ (yellow), $[Cu(dbda)(bcp)]^+$ (red), $[Cu(dbda)(biq)]^+$ (green), $[Cu(dbda)(dap)]^+$ (purple), and N719 (bordeaux). A slight bleaching of the TiO_2 edge near the electrolyte injection hole can be noticed in some samples (b).

Interestingly, after the electrolyte injection in the devices (Figure S13b), the DSSCs based on the dye $[Cu(dbda)(biq)]^+$ (green frame) dramatically changed color from purple to orange after electrolyte injection, suggesting a strong interaction between the dye and the electrolyte components. This phenomenon is currently under investigation, but we hypothesize that the ligand biq may be replaced by other components presents in the electrolyte. The hypothesis is grounded on the study of Randolph P. Thummel *et al.*,[1] who clearly showed that the ligand 2,2'-biquinoline does not bind strongly to the copper center and can be easily replaced by other ligands. The observation made on the complex $[Cu(dbda)(biq)]^+$ before and after the electrolyte injection was easy to make thanks to the significantly red-shifted absorption spectrum of the dye (Figure S12). This tells us that it is critical to consider the binding affinities of the ligands employed for the assembly of Cu(I) complexes and that, although it may not be as evident, we cannot completely rule out the possibility that such an exchange mechanism is occurring to the other complexes in the study. Finally, slight bleaching of the TiO_2 edge near the electrolyte injection hole can be noticed in some samples after the electrolyte injection (Figure S13b). From a simple visual observation, we noticed that the bleaching was less pronounced for the dyes $[Cu(dbda)(dsbtmp)]^+$ and $[Cu(dbda)(bcp)]^+$, which show the highest DSSCs efficiency within the series.

References:

1 Y. Jahng, J. Hazelrigg, D. Kimball, E. Riesgo, F. Wu and R. P. Thummel, *Inorg. Chem.*, 1997, **36**, 5390–5395.
